# PROTOCOL: Aquaculture for improving productivity, income, nutrition and women's empowerment in low‐ and middle‐income countries: A systematic review and meta‐analysis

**DOI:** 10.1002/cl2.1188

**Published:** 2021-07-24

**Authors:** Constanza Gonzalez Parrao, Marta Moratti, Shannon Shisler, Birte Snilstveit, John Eyers

**Affiliations:** ^1^ International Initiative for Impact Evaluation London United Kingdom

## Abstract

The review aims to examine and synthesise the state of the evidence around what works to improve productivity, income, nutrition and women's empowerment outcomes of households involved in aquaculture in low‐ and middle‐income countries. We are particularly interested in addressing the following research questions: (1) Do aquaculture interventions increase the productivity, income, nutrition and empowerment of individuals engaged in aquaculture and their households in low‐ and middle‐income countries? (2) Do aquaculture interventions generate income and nutrition spillover effects beyond the farmers' households? (3) To what extent do the effects of aquaculture interventions vary by intervention type, population group, and location? In particular, to what extent do effects vary by gender? (4) What are the potential barriers and facilitating factors that impact the effectiveness of aquaculture interventions? (5) What is the cost‐effectiveness of different aquaculture interventions focused on productivity, income, nutrition and empowerment outcomes?

## 1 BACKGROUND

### 1.1 The problem, condition or issue

In 2018, global fish production reached a record high of about 179 million tonnes, of which 82 million tonnes, valued at USD 250 billion, came from aquaculture production, which is the farming of aquatic organisms including fish, molluscs, crustaceans and aquatic plants in inland and coastal areas (FAO, 2020a). While global fish production has seen important increases across all continents in the last 20 years, it has almost doubled in Africa and Asia. Over 20 million people are estimated to be engaged on a full‐time, part‐time or occasional basis in aquaculture, making this sector an important source of employment and income across the world. Women account for 19% of this workforce and play a crucial role throughout the aquaculture value chain, providing labour in both commercial and artisanal fisheries (FAO, 2020b).

The growth in aquaculture production has also brought substantial changes in the production systems, raising concerns about the environmental impact of aquaculture and the sustainability of the sector. These detrimental effects include, among others, poor site selection; the use of chemicals and antimicrobials; the impact of escapees on wild stocks; inefficient or unsustainable production of fishmeal and fish oil; or eutrophication (FAO, 2020b; Henriksson et al., 2017). Similarly, the increase and intensification of aquaculture activities can pose a major pressure on land and its use whenever they require converting the use of land into ponds for farming purposes. For example, the shrimp aquaculture sector, successfully established in the 1970–1980s, has been the major cause of mangrove deforestation in Southeast Asia over the last few decades (Richards & Friess, 2016; Valiela et al., 2001). This has been especially controversial since mangroves are an important carbon sink, they support fisheries, provide coastal protection, and their loss and degradation reduce coastal resilience (Barbier et al., 2011; Koh et al., 2018; Mcleod et al., 2011).

To offset these adverse effects and improve governance of the aquaculture sector, the Food and Agriculture Organization of the United Nations (FAO) has championed the Blue Growth Initiative as a framework for a sustainable, economic and social development of fisheries and aquaculture (FAO, 2014a). Examples of practices following this framework include conservation‐oriented management interventions to achieve sustainable coastal aquaculture, implementing protected areas and land zoning to regulate the development of commercial aquaculture, and introducing sectoral innovations, from government support to farmer training and better feeds, to help reduce the environmental footprint of aquaculture (Akber et al., 2020; Henriksson et al., 2017).

Despite the environmental challenges that have arisen from increased production in the sector, aquaculture seems to have great potential to address poverty and nutrition issues, considering that 80% of the world production comes from developing countries (Phillips et al., 2016) and that over 80% of the global aquaculture production is from small‐scale farms that are commonly owned and managed by families (FAO, 2014b). Therefore, in a world of limited resources, aquaculture may have the ability to improve livelihoods and health in developing countries and to contribute to the progress towards a number of inter‐related Sustainable Development Goals (SDGs).

For example, aquaculture could help reduce hunger (SDG 2) and poverty (SDG 1) by making fish available and affordable to combat malnutrition and alleviate nutritional deficiencies (SDG 3: Good health and well‐being). By engaging women into its workforce, aquaculture also has the potential to promote greater equity in access to, and benefits from, economic resources (SDG 5: Gender equality). Finally, aquaculture can contribute to more sustainable development (SDG 14: Conserve and sustainably use the oceans, seas and marine resources for sustainable development) by supporting the production of low carbon footprints among animal source foods (Reale & Phillips, 2020). Thus, well‐planned aquaculture operations could be a key component in sustainable food systems, capable of providing needed animal‐source foods to an increasingly growing population.

Aquaculture is often promoted as a pro‐poor economic activity by acting as a source of income to secure livelihoods for rural populations in low‐ and middle‐income countries (Dey & Ahmed, 2005; Mohamed & Dodson, 1998; Olaganathan & Kar Mun, 2017). However, the scarce empirical evidence around this topic shows a more nuanced picture, in which the impact depends on local production and consumption characteristics of the sector. Recent studies in Ghana (Kassam & Dorward, 2017) and Bangladesh (Rashid et al., 2019) have suggested that aquaculture can have a positive impact on economic growth and poverty reduction at a national level. However, evidence has also highlighted that promoting aquaculture could benefit primarily larger and better‐off farms, thus increasing inequality (Ahmed et al., 1995; Kassam & Dorward, 2017).

The global increase in fish production seems to correspond with a general expansion in fish consumption. The consumption of fish food has increased at an average annual rate of around 3% from the 1960s, a rate higher than all other animal protein foods, and this growth has been observed in both developed and developing countries (FAO, 2020b). Thus, aquaculture has the potential to increase the supply and accessibility of nutritious food that could translate into more nutritious and diverse food diets. Relevant studies have found that agriculture interventions often lead to an increase in food consumption, particularly for the food item targeted by the intervention. Yet the impact of aquaculture on diet quality is more unclear, with evidence being scarce and mixed, often due to the lack of high quality studies and data (Bird et al., 2019; Kawarazuka, 2010; Masset et al., 2012).

Likewise, very little is known about the impact of aquaculture activities on the income, livelihood, nutritional status and health of the women engaged in the sector, and whether aquaculture interventions can promote gender equality and women's empowerment. Women still face significant economic, social and cultural barriers that affect their participation in aquaculture, their access to, and control over assets and resources, and the income and benefits derived from these activities (Johnson et al., 2016; Kruijssen et al., 2018; Morgan et al., 2017; Phillips et al., 2016; Ramírez & Ruben, 2015). The lack of disaggregated data from aquaculture interventions and their evaluations have prevented researchers from capturing important learning for policy and practice, including the ability to assess whether cultural norms reduce or prevent women from reaping the benefits of aquaculture or the circumstances in which the design and implementation of aquaculture interventions can have positive impacts around women's empowerment.

Aquaculture is a sector with potential in several areas of international development, and while there is still limited evidence regarding its impact, synthesising the literature available becomes an increasingly relevant task for programme and policy making. With this review we aim to fill this gap by bringing together existing evidence and exploring, with a gender lens, the impact of aquaculture on productivity, income, nutrition and women's empowerment.^1^

### 1.2 The intervention

The strategic rationale for promoting aquaculture is underpinned by the realisation of expected direct and indirect improvements in development outcomes for individuals, households and communities. Within the review, we will explore aquaculture interventions in low‐ and middle‐income countries that aim to increase productivity, income, nutrition and women's empowerment. We adopt a broad definition of aquaculture, including all types and scales of aquaculture activities to explore its impact along the value chain. We will explore the impact of aquaculture interventions on four broad components: productivity, income, nutrition and women's empowerment.

We follow FAO and refer to aquaculture as the “farming of aquatic organisms including fish, molluscs, crustaceans and aquatic plants in inland and coastal areas. Farming implies some form of intervention in the rearing process to enhance production, such as regular stocking, feeding and protection from predators. Farming also implies the individual or corporate ownership of the stock being cultivated” (FAO, 2020a, p. 23).

In this review, we define “aquaculture interventions” as any project, programme or policy aiming to provide new and/or improved activities at any stage of the aquaculture value chain. Therefore, we will include interventions in all types of aquaculture operations regardless of their scale: from small‐ to medium‐ and large‐scale regarding land size, use of hired labour, capital investment, and level of technological sophistication. In this, we follow Phillips et al. (2016), and acknowledge that definitions based on the scale of the operations are not agreed upon and may have different meanings in different countries and regional contexts. For example, a portion of the literature refers to “small‐scale aquaculture”, referring generally to farming that use low‐input methods and where a large percentage of farm labour is provided by household members. Hence, while we will discuss and analyse definitions and scales of aquaculture operations whenever possible, we aim to map the evidence around the whole sector.

For the review, we will cover different types of aquaculture systems. A key difference exists, for example, between land‐based and water‐based aquaculture. Both systems require access to either land or water bodies, which might represent a barrier to engaging in aquaculture activities, especially when ownership or access is not free or is regulated or precluded to some individuals based on their socioeconomic status. Land‐based systems are more common and usually stock fish in rice fields and ponds on dry land. Water‐based systems involve stocking fish in pens or cages directly in enclosures or attaching them to substrates in coastal or inland waters such as rivers or bays (Halwart et al., 2000). Land‐based aquaculture requires ownership or access to land, while water‐based aquaculture require access to water bodies, which might or might not be free or regulated. When water is accessible, this is often the only aquaculture option for households or individuals with no land or no access to it. Therefore, when access is provided or free, water‐based systems may provide an entry point for landless people and poor fishers to farm fish (Edwards, 2000).

We will include interventions that affect aquaculture along its value chain, covering activities related to input supplies and services, production and postproduction activities, such as processing, trading and marketing.^2^ These interventions are generally productivity‐focused, aiming to improve the quantity and quality of aquaculture production, with the ultimate goal of increasing the income generated from aquaculture activities. However, we will consider aquaculture interventions that improve the efficiency of the sector as a whole and have either a productivity, income or market‐enabling focus. This could involve, for example, providing training or better access to inputs (such as feed, seed and fertilisers), or improving the use and uptake of technology and management practices.

At times, aquaculture interventions aim to combine better aquaculture production and practices with other social and cultural objectives. For example, interventions could also aim to improve community‐based support to aquaculture activities, while others could have additional objectives on nutrition knowledge and practices, or have a deliberate focus on gender equality and empowerment to promote a more equal participation of women in aquaculture and in society. In this review, we will include all types of interventions and highlight when they have any additional social or cultural components. Whenever possible, we will include and look at the impact of aquaculture interventions on productivity, income, nutrition and women's empowerment, as well as the potential additional impact of adding other intervention components on these outcomes. For this purpose, we expect extra components to mostly fit into these two categories:

- Nutrition and behavioural change interventions, which aim to improve awareness and knowledge of the nutritional benefits of healthy diets; for example, emphasising the importance of including fish and other aquatic organisms in diets, especially among pregnant women and children.
- Gender equality and women's empowerment interventions that aim to support and promote women's equal access and participation in the sector.

### 1.3 How the intervention might work

Aquaculture can be a vehicle for improving livelihood and nutrition in low‐ and middle‐income countries. Aquaculture interventions can play a key role in enhancing or accelerating its impact and to ensure the equal distribution of benefits. In this section, we explore four impact pathways through which aquaculture interventions could help deliver benefits along the aquaculture value chains, in terms of productivity, income, nutrition and health, and women's empowerment.

**Figure 1:**
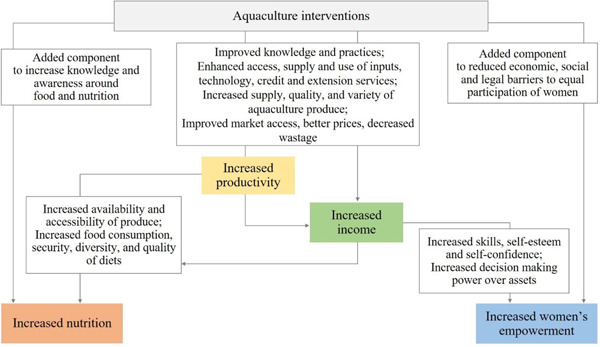
Theory of change

For this review, we use a theory of change that captures the outcomes and mechanisms that apply to a number of generic aquaculture interventions to maintain a clear focus on the key domains: productivity, income, nutrition and empowerment. Figure 1 shows a graphical representation of the theory of change, which distinguishes between main outcomes and intermediary outcomes for these four domains. This section provides a narrative description of the expected pathways to impact, followed by a review of the existing literature on each of them.

The key domains we expect aquaculture interventions to have an impact on is productivity and/or income. Based on Dey and Ahmed (2005), aquaculture production can be increased through at least four pathways: more efficient use of farmers' resources and of existing inputs and technology, the development of new technologies and the transfer of these to farmers, an increase in the use of inputs, and an increase in the area dedicated to fish production. The local environmental and socioeconomic constraints will determine which options are more feasible or likely to be more effective in a specific context, and different aquaculture interventions might therefore focus on one or a combination of the above. Moreover, while interventions might have additional social objectives, we expect the main objective of an aquaculture intervention to be to improve production and productivity within the sector so as to generate and ensure a new or higher source of income and more sustainable livelihood. If this is met, we can then also expect aquaculture to generate positive effects on other domains, such as nutrition and women's empowerment. For example, if productivity of a small fish farmer increases, the farmer can get a higher income by selling more fish to the market or by producing food that ensures better diets for his/her family. When the fish farmer is a woman, and aquaculture generates new or extra skills and income, this can potentially have a positive effect on her self‐esteem, self‐confidence and her role within the household and beyond.

Depending on the specificity of the intervention, productivity and/or income outcomes can be achieved through an increase in some of the following intermediate outcomes: improved access, supply, and use of inputs, technology, credit and extension services or improved aquaculture knowledge and practices, such as better pond management or marketing practices. We can also expect to see an increase in the quantity produced, less waste or an increase in the variety or quality of the aquaculture production. Overall, while interventions might affect these outcomes to a different extent, the ultimate impact will be a more efficient market system, more production, higher productivity and overall a higher return from engaging in aquaculture. This higher return can take different forms: more aquaculture produce to be consumed at home, more income derived from selling aquaculture produce, or more employment opportunities and therefore higher wages in the sector.

The next domain of interest is related to nutrition, addressing how more productivity or income in aquaculture affect nutrition and health of those involved in aquaculture, and if interventions designed with an explicit nutritional component generate a higher impact on nutrition than productivity‐ or income‐focused aquaculture interventions. We expect aquaculture interventions, through increasing production, productivity or income, to make fish and aquaculture more accessible and affordable. This alone could have an impact on food security and on the quantity and quality of nutritious food that household members could enjoy, which in turn, could improve their general health status. This impact will be amplified if the interventions come with additional activities that effectively raise the level of knowledge and awareness on the importance of food and nutrition for health. Whenever behaviour and educational components are incorporated and carried as part of the intervention package, we could expect a higher impact of these nutrition outcomes and on other outcomes such as nutrition knowledge and awareness.

Similarly, if aquaculture interventions affect the level of production, productivity or income of female individuals engaged in the sector, we can expect a positive effect on a number of outcomes related to women's participation and benefits from aquaculture activities, with a potentially positive contribution towards empowerment. Social and cultural norms tend to act as barriers for women and reduce their participation in aquaculture productive activities and eventually the return they get from it. We expect that agriculture interventions, when designed and carried out with a gender equality lens, will help improving the way in which women participate in the sector, the return they get from their participation, and the skills they experience and develop. More opportunities to gain skills and income is more likely to translate into having more productive resources that can help putting women more in control of their decisions, thus improving their roles in their household and beyond. While the ultimate outcome is women's empowerment we appreciate that empowerment is a process as much as an outcome.

#### 1.3.1 Productivity and income

Conceptually, aquaculture interventions that aim to increase production and productivity of aquaculture activities, have both direct and indirect benefits on income, livelihood and poverty. The linkages and pathways are similar to the ones developed in agriculture economics and are discussed extensively for the aquaculture sector (see Ahmed & Lorica, 2002; Rashid et al., 2019; Toufique & Belton, 2014). For example, Toufique and Belton (2014) define the following four linkages: direct consumption links (increased consumption from own production), indirect consumption links (increased availability and accessibility of fish), direct income links (increased income for aquaculture producers), and indirect income links (employment in the fish value chain and consumption linkages).

The income linkage is based on the assumption that aquaculture interventions, by improving efficiency along the value chain, can generate higher return and therefore higher incomes for the farmers involved. Some interventions will affect more specifically the productivity side of aquaculture operations, while others will focus on the aquaculture market. We expect most interventions to be productivity‐focused and affect income via an increase in production and productivity; however, some market‐oriented interventions may also affect revenues and income directly, not necessarily via productivity, and we specifically allow this pathway in our theory of change. Either way, we expect an impact on individuals and households involved, and if aquaculture engages poor households, this could have a direct impact on their incomes and on their poverty status. Moreover, aquaculture growth can have an impact on employment opportunities, and more generally on economic growth, thus benefiting communities beyond the individuals engaged in aquaculture.

From a consumption side, increase in availability and accessibility of aquaculture produce might have an impact on prices, which will affect the consumers' ability to buy fish and other aquaculture produce (whether they are producers or not) and, thus, increase real incomes. The overall impact on the economy and poverty would be an empirical matter and will depend on who are the aquaculture producers (poor vs. nonpoor), who consumes fish and how consumption responds to possible changes in prices, and to the overall magnitude of the direct and indirect effects on the economy and poverty.

Studies highlight how the distributional impact of aquaculture could even be negative if the poor cannot rip the benefits of aquaculture or if the benefits are mostly concentrated in the hands of few large better‐off producers. For example, whenever aquaculture requires a minimum level of access to land, technology and resources, the poorest, often landless households, will not be likely to benefit from it. Thus, the promotion of aquaculture would benefit larger and better off farms, increasing inequality (Ahmed et al., 1995; Kassam & Dorward, 2017).

Empirical studies that help quantify the specific linkages and provide an overall impact of aquaculture interventions on income and poverty of different types of households are still quite limited. Other studies have often found correlations between aquaculture activities and poverty, but it is harder to make attribution claims if studies are not designed with the specific objective of assessing the impact of aquaculture on the overall consumption and welfare status. A few examples of empirical studies include Kassam and Dorward (2017), who investigated the poverty impacts of pond and cage aquaculture in Ghana, and Rashid et al. (2019), who analysed aquaculture production and its impact on prices, consumption, income for different types of households in Bangladesh.

Interestingly, both studies found that aquaculture had a positive impact on the economy and contributed to a reduction in poverty levels in their countries: Kassam and Dorward (2017) found that the overall impact occurred mostly via the indirect effects on economic growth of nonpoor farmers, while Rashid et al. (2019) found that an increase in production benefited all producers (who are both poor and nonpoor) and that the reduction in prices benefited all population, in particular poorer households, thus generating a substantial impact on the country's poverty level.

Kassam and Dorward (2017) aimed to assess the poverty impacts of small‐scale pond aquaculture and small‐medium enterprises (SME) cage aquaculture in Ghana, and to compare the relative significance of the direct impacts on poor small‐scale fish farmers and the indirect impacts on economic growth and employment from SMEs. They found that nonpoor small‐scale pond fish farmers who have been trained and/or use better management practices hold the most potential to impact poverty indirectly through generating economic growth. These indirect impacts are higher than the direct impacts on poor small‐scale fish farmers and the indirect impacts from SMEs.

Rashid et al. (2019) found that the impacts of aquaculture growth on income distribution and poverty reduction in Bangladesh have been substantial, with aquaculture explaining almost 10% of the overall poverty reduction in Bangladesh during the first decade of the 21st century. Bangladesh experienced a rapid growth in the demand of aquaculture fish since 1980s, but its supply increased even more rapidly, resulting in a decline in real price. The growth in production led to higher incomes for producers but also lower prices for consumers, which includes to some degree the producers as they also consume fish. This in turn translated into increased consumption for all types of households, in particular for the bottom two income quintiles, income gains for all households, particularly in aquaculture producers, and an overall substantial reduction in the proportion of households below the poverty lines.

Overall, the literature suggests that while aquaculture is often promoted as a propoor economic activity with high potential impact for the poorest households, the empirical evidence is quite scare and the picture more nuanced, with the impact depending on the specific characteristics of the production and consumption patterns of the sector. More quality studies and evaluations of aquaculture interventions are needed to help inform how the income and poverty impact can be promoted effectively and equitably.

In this systematic review, we will bring together studies that explore how aquaculture interventions affect production, productivity, income, market and prices. We would like to explore how effective aquaculture interventions are, for whom they work best at increasing the use of technology, quantity, quality and variety of aquaculture produce, and the overall improvement in skills and practices.

#### 1.3.2 Nutrition, health and food security

Whenever aquaculture interventions succeed to promote greater quantity or higher quality aquaculture production that translates into better quality consumption, we can expect an impact on nutrition and food security among individuals engaged in aquaculture and, more generally, for the entire country. Conceptually, the impact pathways on nutrition can occur via two main mechanisms. First, an increase in quality of diets can occur due to an increase in own consumption when aquaculture farmers produce more quantity and quality of nutritious food and keep some of it for their personal consumption. Second, an increase in the consumption of nutritious food from aquaculture could occur as a result of an increase in real incomes. Higher incomes from aquaculture could lead to more resources to buy more or better food at the market and, therefore, have an impact on nutrition and quality of diets.

The impact on nutrition via the second mechanism affects all households in a community, whether they are involved or not in aquaculture. If aquaculture interventions lead to more accessible aquaculture produce in the economy, real incomes increase even for households not engaged in aquaculture. Hence, all consumers could afford a more nutritious food basket and receive the associated dietary benefits.

The link between higher income and nutrition is well‐established in the literature and earlier studies on agriculture identified that increasing household income is a particularly important factor to improve dietary intake, as the consumption of nonstaple foods is positively related to increases in income (Hawkes & Ruel, 2006; Leroy & Frongillo, 2007; World Bank, 2007). Though there is a paucity of research on the impact of aquaculture on nutrition, useful insights can be drawn from the broader agriculture literature, which sometimes also includes aquaculture interventions. Studies tend not to be able to separate out the two mechanisms and measure the overall effect on the consumption.^3^

Relevant studies on nutrition have found that agriculture can lead to an increase in consumption, in particular for the food item targeted by the intervention, but the impact on nutrition is more unclear. Ruel and Alderman (2013) used a similar framework to our review when examining the literature on home gardens and homestead food production systems. The authors found that there is little evidence of effectiveness of homestead food production programmes on maternal or child nutrition status (i.e., anthropometry or micronutrient status), with the possible exception of vitamin A status. Moreover, they found that the nutritional effect is more likely when agriculture interventions target women and include women's empowerment activities, such as improving their knowledge and skills through behaviour‐change communications or promoting their increased control over income from the sale of targeted commodities.

In addition, a review by Masset et al. (2012) of the impact of agriculture interventions (mostly home gardens) on nutrition found that most studies reported a positive effect on food composition. Depending on the interventions, they found an increase in the consumption of the food item targeted by the intervention (more fish consumption for aquaculture interventions, more dairy products for dairy interventions, and so forth) but little evidence was available on changes in the diet, micronutrients' intake, and children's nutritional status. Similarly, Bird et al. (2019) reviewed the impacts of agriculture interventions on nutritional outcomes in South Asia and found no convincing evidence of an impact of agricultural interventions on child anthropometric measurements. One study included in the review (Pant et al., 2014) looked specifically at the impact of aquaculture interventions on nutrition in Bangladesh. The authors found that, compared to baseline, households increased their monthly consumption of fish, meat and eggs, and increased annual household income. Similar increases in consumption were found by Kawarazuka (2010), who looked specifically at the impact of pond‐based aquaculture on dietary intake/nutritional status.

Taken together, these studies suggest that agriculture interventions can lead to more consumption, especially for the food item targeted by the interventions. However, this increased consumption might or might not translate into a measurable impact on nutrition. Masset et al. (2012) attribute the lack of evidence on nutritional status to the methodological weaknesses of the studies reviewed, rather than to a lack of impact, and calls for more research on the topic.

With this review, we will bring together and analyse the studies that look specifically at aquaculture with the aim to shed some light on whether and how aquaculture interventions can be effective at promoting better quality food consumption that translates into better nutrition and health.

#### 1.3.3 Aquaculture and women's empowerment

SDG5 puts gender equality and empowerment of women and girls on top of the development agenda. Women should enjoy full and effective participation and equal opportunities at all levels of decision making in political, economic and public life and man and women should have equal rights to benefit from economic resources.

The extent to which aquaculture interventions contribute to empower women and girls is unclear.^4^ Conceptually, to the extent that aquaculture engages women in new and/or more productive economic activities, aquaculture has the potential to expand their choice, strengthen their voice and increase the importance and role of women within the household and the communities. Aquaculture could provide a means for women to generate more income for themselves and their families, as well as acquire and develop knowledge and skills. This could lead to having more voice, respect and control over her and her household decisions.

Johnson et al. (2018) provide a useful framework to distinguish between impacts of interventions on female empowerment and identify three main approaches: reaching women, benefitting women, and empowering women. An intervention focusing on reaching women emphasises engaging women in project activities and tracks progress in terms of participation, for example measuring the number of women who attend meetings or receive training. In an intervention focused on benefitting women, the focus is on ensuring that the outcomes the project is seeking—for example, reduced hunger, increased income or greater resilience—are captured by women. Empowering women involves strengthening their ability to make strategic life choices and to put those into action.

Evidence from agriculture show that even when interventions lead to improvements in women's agricultural production, income or nutritional status, they rarely succeed in reducing underlying inequities between men and women (Johnson et al., 2016, 2018; Quisumbing et al., 2013; Santos et al., 2014). Following Johnson et al. (2018) framework, while increasing the income that women earn would be considered “benefiting” women, if women do not have increased control over how this income is managed or used, an intervention would not be “empowering” women.

Despite the importance of the sector, and the interest around what works to promote women's empowerment, the literature on aquaculture and gender is scarce. Evidence is limited on the quality of female participation and the economic returns from aquaculture. Additionally, the lack of sex‐disaggregated data is an issue often highlighted in the literature as it reduces the potential for gender analysis of the sector, which is the basis for the development of gender sensitive policies and planning (FAO, 2014a, 2020b; Harper et al., 2013; Kruijssen et al., 2018; Weeratunge et al., 2010).

Economic, social, and cultural barriers affect the participation of women to the sector, their access and control over assets and resources, and the income and benefits they derive from the activities they perform (Johnson et al., 2016; Kruijssen et al., 2018; Morgan et al., 2017; Ramírez & Ruben, 2015). Below we discuss some of these barriers and, more generally, the social norms and cultural dynamics that affect women's position in the sector.

Kruijssen et al. (2018) put together the most comprehensive review on aquaculture and gender to date and find gendered imbalances along different dimensions (including division of labour, distribution of benefits, access and control over assets and resources, gender and social norms, power relations and governance), arguing that these formal and informal barriers, including gender norms, would limit women's equal engagement and returns. In addition, women face unequal access to aquaculture as they tend to have less access and control over assets, including a disadvantage in ownership and control of land or ponds (Ndanga et al., 2013; Veliu et al., 2009). For example, female farm ownership is 2%–3% in Vietnam (Veliu et al., 2009), female pond ownership is <1% in Bangladesh (Khondker et al., 2010), and women tend to have less access and control over capital (Ndanga et al., 2013), skills, technologies and extension services (Morgan et al., 2017).

When women participate in aquaculture labour activities, their roles vary significantly across countries and production nodes, so it is not appropriate to generalise; however, benefits they get are often less than their male counterparts. Nevertheless, FAO (2020b) highlights that women play an important role throughout the value chain, providing labour in both commercial and artisanal fisheries and identifies small‐scale production, postharvest industrial and artisanal processing, value addition, marketing and sales as the most common roles for women in aquaculture. Evidence suggests that women tend to receive lower returns and are disproportionately represented in less‐profitable nodes of aquaculture value chains (Kruijssen et al., 2013) or where jobs are regarded as especially insecure (Kruijssen et al., 2018; Veliu et al., 2009). For example, a case study on Cameroon found that women find it challenging to combine domestic workload with aquaculture activities and prefer activities that could be undertaken in evenings or in spare moments over those that required dedicated, daily supervision (Brummett et al., 2011). In Kenya, when fish processing became profitable, men replaced women who first had those jobs (Ndanga et al., 2013). Lastly, a study from Chile showed that women faced no cultural barriers to their entry in the growing aquaculture job market; however, access to jobs in the sector did not come with equal returns and the study found salary differences in favour of men, as a result of gender discrimination (Ramírez & Ruben, 2015).

Overall, evidence suggests that social norms and cultural dynamics significantly affect and shape women's participation and return from aquaculture (Morgan et al., 2017; Ramírez & Ruben, 2015), affecting women's capacity to adopt and retain aquaculture technologies (Morgan et al., 2017) or to translate economic returns into more empowerment (Sari et al., 2017). In Bangladesh, one study found key gender differences in the division of labour, in the levels of decision‐making power, and in access to and control over resources and benefits from aquaculture, identifying that these differences are rooted in and perpetuated by social and gender norms and relations (Kruijssen et al., 2016).

In order for aquaculture interventions to have any effect on improving gender equity or promoting empowerment, they need to take into account the specific social norms of the context they operate in and the barriers they create for women. Interventions need to be targeted and realise the importance of addressing underlying social and gender norms. While addressing underlying social and gender norms is likely to be beyond the aim of any individual aquaculture intervention, positive contributions in this direction can be made through awareness training and community support, giving explicit attention to gender‐based constraints, access and control over resources, decision‐making power, and gender norms (Kruijssen et al., 2016; USAID, 2013).

### 1.4 Why it is important to do this review

There has been an advocacy for aquaculture research and production guidelines for decades (Pullin & Shehadeh, 1980). Aquaculture production has continued to develop since, reaching a record high in 2018 after having doubled in the past 20 years in Asia and Africa. More importantly, aquaculture is projected to supply more than half of the world's fish‐based food by 2030, and then take over future fish sourcing (World Bank, 2013).

This steady increase in production has been in line with investment and research efforts from government agencies, international organisations and academic centres, which have continued to promote aquaculture as a sustainable option to feed the world's growing population. The following are examples of recent aquaculture programmes that reflect the extent of these efforts.

The Global Environment Facility (GEP) provides funding to developing countries and countries with economies in transition to help them meet the objectives of international environmental conventions. In the last 5 years, GEP has supported government programmes in Bangladesh, Chile, Malawi, Myanmar and Timor Leste to make their aquaculture activities more climate change resilient, adding up to almost USD 23 million (GEP, n.d.).

In 2012, the Aquaculture for Food Security, Poverty Alleviation and Nutrition (AFSPAN), an EU‐funded, 3‐year project coordinated by FAO was created to understand the link between aquaculture and food security. With a EUR one million budget, the project was implemented in 11 developing and low‐income, food‐deficit countries. AFSPAN concluded that aquaculture contributes significantly to food security and nutrition, as well as to other outcomes such as job creation, income generation, and women's empowerment (CORDIS, 2015).

Under the Feed the Future multiyear strategy, the United States Agency for International Development has supported two aquaculture programmes in Bangladesh. The first project, Aquaculture for Income and Nutrition (AIN), was implemented by WorldFish between 2011 and 2016 with a USD 25 million budget. AIN aimed to increase aquaculture quality production, improve the nutrition and income status of farm households, promote commercial aquaculture, and support capacity building of the public and private sector (Keus et al., 2017). Building on the success of AIN, a second programme is being implemented, the Bangladesh Aquaculture and Nutrition Activity. Starting in 2018, this 5‐year and USD 24.5 million project intends to develop a more inclusive sector by strengthening the aquaculture market systems and a nutrition‐based behaviour with special focus on women and youth (WorldFish, n.d.).

The increase in aquaculture production and fish‐based food consumption, coupled with the challenges that climate change is posing to the sustainability of our diets, to which aquaculture might represent a solution, provide a timely backdrop for an up to date review of the impact of aquaculture interventions on productivity, income, nutrition and women's empowerment to contribute to policy and programming in the sector.

In turn, while there is some relevant literature on agriculture and its impact on nutrition, few quality studies exist, specifically on aquaculture. Moreover, despite the increasing importance of aquaculture, to our knowledge no effort has been made to draw insights from how best to design and implement aquaculture interventions when income, nutrition and women's empowerment are the key objectives.

There are a number of relevant existing reviews. Our review differs in two ways: first, it will be the first review with a specific focus on aquaculture interventions. Second, we will explore the literature from a gender lens. Previous reviews, detailed below, looked at either the broader agricultural sector, which included none or only few aquaculture interventions (Bird et al., 2019; Masset et al., 2012; Ruel et al., 2018) or covered aquaculture under a narrow scope (D'Armengol et al., 2018; Gambelli et al., 2019).

The systematic review led by Bird et al. (2019) looked at peer‐reviewed studies published between 2012 and 2017, detailing impacts of household‐ or farm‐level agricultural interventions on nutritional outcomes in South Asia. The authors identified six intervention studies and found mixed evidence of impact. Interventions had a positive impact on intermediate outcomes on the pathway from agricultural intervention to nutritional or health status, including dietary quality and dietary diversity of households and individuals. The evidence on the impact on final nutritional outcomes was mixed: one paper reported that home gardens with poultry reduced the odds of anaemia, but there was no convincing evidence of an impact of agricultural interventions on child anthropometric measurement, as reported in four papers.

Masset et al. (2012) conducted a systematic review of the evidence around effectiveness of agricultural interventions (including biofortification, home gardens, small scale fisheries and aquaculture, dairy development, and animal husbandry and poultry development) aiming at improving the nutritional status of children. The review included 23 studies, mostly evaluating home garden interventions. The authors found that the interventions had a positive effect on the production of the agricultural goods promoted, but not on households' total income. The interventions were successful in promoting the consumption of food rich in protein and micronutrients, but the effect on the overall diet of poor people remains unclear. The evidence reviewed showed no effect of these interventions on nutritional status of children, but methodological weaknesses of these studies cast serious doubts on the validity of the results. The authors attribute this to the lack of statistical power of the studies reviewed rather than to the lack of effectiveness of the interventions.

Ruel et al. (2018) reviewed the evidence related to nutrition‐sensitive agriculture programmes from 2014 onwards, including 16 impact evaluations and 28 observational studies. The authors found that all programmes were highly successful at both meeting their production and consumption targets, and at providing households with access to nutrition‐rich foods. However, none of the impact evaluations identified in the review covered aquaculture interventions.

On the other end of the spectrum, some reviews had a narrow scope that shed lights on specific aspects of the aquaculture sector. d'Armengol et al. (2018) focused particularly on small‐scale fisheries with a comanagement structure and component. The authors included 70 studies and found that comanagement delivers both ecological and social benefits, as it increases the abundance and habitat of species, fish catches, actors' participation, and the fishery's adaptive capacity, as well as induces processes of social learning. In turn, Gambelli et al. (2019) brought together studies in the field of the economic dimension of organic aquaculture. The authors found that profitability in organic aquaculture is not guaranteed for all aquaculture species, and that the feed and other fixed costs can be an issue if these are not balanced by adequate price premiums.

Moreover, while none of the existing reviews explored the impact on aquaculture from a specific gender perspective, one review focused on gender issues in aquaculture. Kruijssen et al. (2018) reviewed the evidence on gender relations in aquaculture value chains by looking at the gender division of labour, distribution of benefits, access and control over assets and resources, gender and social norms, and the power relationships within and outside the chain. The review showed that there is limited high quality sex‐disaggregated data regarding aquaculture value chains. Existing evidence, however, indicates gendered imbalances in all the dimensions assessed, with women's equal engagement and returns being limited by formal and informal barriers.

With the present review, we intend to provide an up to date review of existing evaluation studies that explore the impact of aquaculture interventions on productivity, income, nutrition and women's empowerment to fill the existing gaps on impact of aquaculture and its gender dynamics.

## 2 OBJECTIVES

The review aims to examine and synthesise the state of the evidence around what works to improve productivity, income, nutrition and women's empowerment outcomes of households involved in aquaculture in low‐ and middle‐income countries.

We are particularly interested in addressing the following research questions:

1. Do aquaculture interventions increase the productivity, income, nutrition and empowerment of individuals engaged in aquaculture and their households in low‐ and middle‐income countries?
2. Do aquaculture interventions generate income and nutrition spillover effects beyond the farmers' households?
3. To what extent do the effects of aquaculture interventions vary by intervention type, population group, and location? In particular, to what extent do effects vary by gender?
4. What are the potential barriers and facilitating factors that impact the effectiveness of aquaculture interventions?
5. What is the cost‐effectiveness of different aquaculture interventions focused on productivity, income, nutrition and empowerment outcomes?

## 3 METHODS

For this review, we will follow the Methodological Expectations of Campbell Collaboration Intervention Reviews (MECCIR) Conduct and Reporting Standards (2019a, 2019b) and our process will be based on recognised guidelines for systematic reviews of effectiveness in international development (Waddington et al., 2012).

To address research questions 1–3, we will synthesise evidence provided in impact evaluation studies and, whenever possible, analyse its corresponding effect size data. This will allow us to provide estimates of average effects and heterogeneity of reported changes in outcomes measured within each of the pathways described in the theory of change.

To capture evidence on the context, implementation and underlying mechanisms, we will also adopt a mixed‐methods, theory‐based approach to address research question 4. Under the “effectiveness+” framework (Snilstveit, 2012), we will search and synthesise supplementary evidence, including information derived from intervention documents, process evaluations, formative assessments or similar documentation.

Finally, to address research question 5, we will search and synthesise cost data for the interventions of interest drawing on standard approaches to synthesise economic appraisal evidence (Shemilt et al., 2008). If available, these data will inform policy and decision makers about the relative cost‐effectiveness of different types of aquaculture interventions, as described below.

### 3.1 Criteria for considering studies for this review

#### 3.1.1 Types of studies

To address research questions 1–3, we will include evaluations that use an experimental or quasi‐experimental design to robustly measure a change in outcomes that is attributed to an intervention as is compared to an appropriate counterfactual. We will include randomised studies and nonrandomised studies as described below.

##### Randomised controlled trials (RCTs)

- RCTs, with assignment at individual, household, community or other cluster level, and quasi‐RCTs using prospective methods of assignment such as alternation.

##### Nonrandomised studies

- Regression discontinuity designs, where assignment is done on a threshold measured at pretest, and the study uses prospective or retrospective approaches of analysis to control for unobservable confounding.
- Studies using design or analytical methods to control for unobservable confounding, such as natural experiments with clearly defined intervention and comparison groups, which exploit natural randomness in implementation assignment by decision makers (e.g., public lottery or random errors in implementation), and instrumental variables estimation.
- Studies with pre‐ and postintervention outcome data in intervention and comparisons groups, where data are individual level panel or pseudo‐panels (repeated cross‐sections), which use the following methods to control for confounding:
  − Studies controlling for time‐invariant unobservable confounding, including difference‐in‐differences, or fixed‐ or random‐effects models with an interaction term between time and intervention for pre‐ and postintervention observations.
  − Studies assessing changes in trends in outcomes over a series of time points (e.g., interrupted time series [ITS]), with or without contemporaneous comparison (e.g., controlled ITS), with sufficient observations to establish a trend and control for effects on outcomes due to factors other than the intervention.

- Studies which control for observable confounding, including nonparametric and parametric approaches:
  − Nonparametric approaches, for example, statistical matching, covariate matching, coarsening, propensity score matching.
  − Parametric approaches, for example, propensity‐weighted multiple regression analysis.

While we will also consider evaluations of pilot studies aimed to be scaled up, efficacy studies, feasibility studies, acceptability studies, literature reviews and systematic reviews will not be included as primary studies.

To address research question 4, we will include a broader range of evidence, if available, to provide a better understanding of the intervention design, implementation, context and intended or unintended mechanisms. This information could be sourced from design documents, monitoring and evaluation reports, and other documentation related to the implementation of the interventions of interest.

To assess the relative cost‐effectiveness of interventions from included studies, as stated in research question 5, we will consider relevant documentation on these economic evaluations. This could include evidence on unit or total costs to implementers, participants and nonparticipants as relevant, with the aim to compare data across interventions.

#### 3.1.2 Types of participants

The unit of analysis for this review may be individuals, households, villages, municipalities or community‐based organisations. The study sample will be based in low‐ and middle‐income countries in accordance with widely used international classifications (World Bank, n.d.). We anticipate that studies will mainly focus on people living in rural areas; however, studies in which participants live in periurban or urban areas will also be eligible. Participants may be of any age, and there will be no restrictions based upon any other demographic characteristics.

#### 3.1.3 Types of interventions

To understand potential differences between aquaculture interventions and to capture the role of women across these activities, we will have a broad definition of interventions. We will include any project, programme or policy that seeks to provide new and/or improved aquaculture activities in any of the various stages of its value chain, including input supplies and services, production, processing, trading or marketing. For example, this could include activities related to farming fish and other aquatic organisms (e.g., seaweed), based on ponds, cages, and other aquaculture systems, involving land‐based and water‐based aquaculture for which there is relevant evidence.

The majority of aquaculture production activities are conducted by small scale farms, owned or managed by families (FAO, 2014b). Hence, we anticipate that included studies will focus on smallholder farming interventions. However, we will not exclude studies if their focus is on larger scale aquaculture activities.

Finally, for the review we will include any type of programme that promotes aquaculture in low‐ and middle‐income countries, which might also include one or a combination of aquaculture efficiency‐focused interventions, behavioural change interventions, capacity and skill development interventions, and gender equality and women's empowerment interventions.

#### 3.1.4 Types of outcome measures

##### Primary outcomes

To address research questions 1–3, we will focus on four groups of primary outcomes: productivity, income, nutrition, and empowerment. Because the scope for the review is rather broad, the description of these groups, presented below, is not exhaustive and represents only examples of how these outcomes could be measured in our set of included studies.

The first group of outcomes relates to the production, productivity, and market aspects of aquaculture activities. Examples of this group include prices of aquaculture production, measures of supply, accessibility and quality of inputs (such as seeds or fertiliser), access to markets, use of technology, or management practice.

The second group relates to the income of individuals engaged in aquaculture and their households. This would include examples such as the amount of income derived aquaculture activities, the ratio of income derived from aquaculture on the total income, and consumption expenditure measured at the individual or household level. Other relevant welfare outcomes could refer to poverty (using income or consumption poverty measures) or other multidimensional poverty or livelihood measures.

The third group, nutrition outcomes, relates to quantity, quality and diversity of the diet and health status of the participants and their households. The literature often measures these outcomes using food consumption levels or, to better capture quality, food security or food diversity scores, such as the Household Dietary Diversity Score (Swindale & Bilinksy, 2006). Nutrition measures include anthropometric measures, such as body mass index (BMI) for adults and weight‐for‐height, height‐for‐age and weight‐for‐age for children. Additionally, we would also be interested in changes in knowledge and awareness on nutrition and quality of diets, and other health related indicators.

The fourth group of outcomes is related to the empowerment of women engaged in aquaculture activities. These measures generally look at whether and to what extent women have control over a number of dimensions as a proxy for their empowerment and control over their lives, including income from aquaculture (from an involvement in any of the stages of its value chain), household consumption and spending decisions. Outcomes for this group could also include measures of confidence and trust in the community, equal participation along the aquaculture value chain, reduced wage gap, changes in attitude towards women, or established tools such as the Women's Empowerment in Agriculture Index (IFPRI, 2012).

##### Secondary outcomes

We will map all other outcomes measured in our set of included studies if these cannot be categorised within the main four groups of primary outcomes. While at this stage we cannot predict all potential secondary outcomes, examples might include environmental or social measures outside the aquaculture value chain but associated to aquaculture activities. If any adverse effects are reported, we will include these outcomes as well.

#### 3.1.5 Additional criteria

We will search for relevant studies using the following additional criteria. We will include studies published in any language, although we will develop search terms in English. Considering the intervention types and study designs defined for the review, we do not expect to identify relevant studies before 1980; hence, we will include studies with publication dates of 1980 or after. To minimise the potential of publication bias, we will include studies regardless of their publication status; this covers studies identified in academic journals, books, institutional reports, conference proceedings, theses and dissertations or organisational websites. We will include studies with any length of follow‐up periods. Finally, we will only include studies focused on low‐ and middle‐income countries; however, we do not anticipate imposing any additional setting restrictions for the review.

To exemplify the criteria described above, our scoping work has identified studies that are (un)likely to meet our criteria, and hence, would be included and excluded following the review framework:

*Included*

- Haque and Dey (2017)
- Rand and Tarp (2009)
- Saiful Islam et al. (2015)

*Excluded*

- Dey and Ahmed (2005): this article provides an overview of technological and policy issues to consider in aquaculture; hence, while its topic is relevant, it does not focus on the evaluation of a relevant intervention.
- Mohamed and Dodson (1998): this article provides a needs assessment and a pilot evaluation of an aquaculture project based on data from in‐depth interviews. Therefore, it is not aligned with the type of studies considered for the review.
- Olaganathan and Kar Mun (2017): this article reviews relevant literature to summarise the impacts of aquaculture on livelihood and food security of rural communities. While this is not the type of study we would consider for the review, we would screen its list of references to identify potentially relevant studies.

### 3.2 Search methods for identification of studies

#### 3.2.1 Electronic searches

We will search for relevant studies on the following academic databases, organisational repositories, and agencies websites. To reduce the risk of publication bias, these information sources were selected to cover a range of publication types, including journal articles, working and discussion papers, conference proceedings, thesis and dissertations, and institutional reports. The review team will document the literature search process, including the search strategies adapted for each source.

##### Academic databases

- 3ie Development Evidence Portal: https://developmentevidence.3ieimpact.org
- British Library for Development Studies: https://guides.lib.sussex.ac.uk/c.php?g=655545&p=4613793
- EBSCO (Agricola, AGRIS, CAB Abstracts^5^, Gender Studies Database, GreenFILE, IDEAS‐Repec, World Bank eLibrary): www.ebsco.com
- Econlit (Ovid): www.ovid.com/site/catalog/databases/52.jsp
- Scopus: www.scopus.com

##### Grey literature sources

- African Development Bank Group (AfDB): www.afdb.org/en/documents/publications
- Asian Development Bank: www.adb.org/what-we-do/data/publications
- CARE International: www.careevaluations.org
- Consultative Group on International Agricultural Research (CGIAR): https://cgspace.cgiar.org/handle/10568/83389
- ELDIS, Institute of Development Studies: www.eldis.org
- Food and Agricultural Organisations of the United Nations (FAO)—Fisheries and Aquaculture Department: www.fao.org/fishery/publications/search/en
- Foreign, Commonwealth and Development Office (FCDO): www.gov.uk/research-for-development-outputs
- Global Environmental Facility (GEF): www.gefieo.org/evaluations/all?f%5b0%5d=field_ieo_grouping%3A312
- Innovations for Poverty Actions (IPA): www.poverty-action.org/search-studies
- Inter‐American Development Bank (IDB): https://publications.iadb.org/en
- International Food Policy Research Institute (IFPRI): www.ifpri.org/publications
- International Fund for Agricultural Development (IFAD): www.ifad.org/en/web/ioe/evaluations
- J‐Poverty Action Lab (J‐PAL): www.povertyactionlab.org/evaluations
- OXFAM International: https://policy-practice.oxfam.org.uk/publications
- Overseas Development Institute (ODI): www.odi.org/publications
- Registry for International Development Impact Evaluations (RIDIE): https://ridie.3ieimpact.org
- Search4DEV: www.bibalex.org/Search4Dev/Category/subject
- United States Agency for International Development (USAID): www.usaid.gov/reports-and-data
- WorldFish: www.worldfishcenter.org/search/publications
- World Food Programme (WFP): www.wfp.org/publications
- World Health Organisation (WHO): www.who.int/publications

#### 3.2.2 Searching other resources

While systematic reviews and narrative literature review are not eligible for inclusion, we will screen the reference lists of relevant reviews. These could be identified by the search strategy or by the research team. Likewise, we will screen the reference lists of all included studies. Lastly, using Google Scholar, we will also conduct a forward citation tracking for all included studies.

Additionally, we will conduct a second search of references to address research questions 4 and 5 regarding factors that hinder or facilitate the effectiveness of aquaculture interventions and a cost‐effectiveness analysis of such interventions. This search will focus on information related to the interventions covered by the included studies, in the form of supplementary documents, studies or reports including contextual information, cost data, process evaluations or similar documentation. We will undertake this search using Google and based on the intervention name.

Once the screening process concludes and we have the list of included studies, we will contact the review's advisory group and publish a public note (i.e., an institutional blog listing our included studies) to try to identify additional records, either as included studies or as contextual documents of included interventions. We will make every effort to contact authors from included studies to locate further contextual information as needed.

### 3.3 Data collection and analysis

#### 3.3.1 Description of methods used in primary research

Using the inclusion criteria set out in the previous sections, we anticipate that primary studies included in this review will use experimental or quasi‐experimental study designs and/or analysis methods to examine the extent to which changes in outcomes are attributable to the intervention. To this end, we will include randomised studies as well as nonrandomised studies that are able to suitably account for selection and confounding bias (Waddington et al., 2017).

#### 3.3.2 Criteria for determination of independent findings

Complex data structures are a common occurrence in meta‐analyses of impact evaluations. There are several scenarios through which these complex structures with dependent effect sizes might occur. For instance, there could be several publications that stem from one study, or several studies based on the same data set. Some studies might have multiple treatment arms that are all compared to a single control group. Other studies may report outcome measurements from several time points, or use multiple outcome measures to assess related outcome constructs. All such cases yield a set of statistically dependent effect size estimates (Borenstein et al., 2009).

The research team will assess the extent to which relationships exist across the studies included in the review. We will make every attempt to avoid double counting of identical evidence by linking papers before data analysis. Where we have several publications reporting on the exact same effect, we will use effect sizes from the most recent publication. We will utilise information provided in studies to support these assessments, such as samples sizes, programme characteristics and key implementing and/or funding partners.

We will extract effects reported across different outcomes or subgroups within a study, and where information is collected on the same programme for different outcomes at the same or different periods of time, we will extract information on the full range of outcomes over time. Where studies report effects from multiple model specifications, we will use author's preferred model specification. If this is not stated or is unclear, we will use the specification with the most controls. Where studies report multiple outcome subgroups for the same outcome construct, we may calculate a “synthetic effect size” (Borenstein et al., 2009, ch. 24). Where studies report multiple outcomes or evidence according to subgroups of participants, we will record and report data on relevant subgroups separately. Further information on criteria for determining independent effect sizes is presented below.

We will deal with dependent effect sizes in one of two ways, either through the use of robust variance estimation (RVE: Fisher & Tipton, 2015; Hedges et al., 2010), or through data processing and selection techniques. RVE using a small sample adjustment will be the preferred analytic method when feasible. The RVE approach allows us to use all available data in our effect size estimates, even data that is statistically dependent. However, these analyses must have >4 degrees of freedom to make valid inferences. In cases where analyses do not meet this criteria, data processing and selection techniques will be used to deal with dependent effect sizes.

If RVE analyses are not feasible for a meta‐analysis of any given intervention or outcome group, we will utilise several criteria to select one effect estimate per study. Where we have several publications reporting on the same study, we will use effect sizes from the most recent publication. For studies with outcome measures at different time points, we will follow De La Rue et al. (2013) and synthesise outcomes measured immediately after the intervention (defined as 1–6 months) and at follow‐up (longer than 6 months) separately. If multiple time points exist within these time periods, we will use the most recent measure. We anticipate many of the interventions we include in our review will be ongoing programmes and the follow‐up will, therefore, reflect duration in a program rather than time since intervention. When such studies report outcome measures at different time points, we will identify the most common follow‐up period and include the follow up measures that match this most closely in the meta‐analysis. When studies include multiple outcome measures to assess related outcome constructs, we will follow Macdonald et al. (2012) and select the outcome that appears to most accurately reflect the construct of interest without reference to the results. If studies include multiple treatment arms with only one control group and the treatments represent separate treatment constructs, we will calculate the effect size for treatment A versus control and treatment B versus control and include in separate meta‐analyses according to the treatment construct. If treatments A and B represent variations of the same treatment construct, we will calculate the weighted mean and SD for treatment A and B before calculating the effect size for the merged group versus control group, following the procedures outlined in Borenstein et al. (2009, ch. 25). Where different studies report on the same programme but use different samples (e.g., from different regions) we will include both estimates, treating them as independent samples, provided effect sizes are measured relative to separate control or comparison groups.

#### 3.3.3 Selection of studies

We will begin by importing all search results into EPPI‐Reviewer 4 (Thomas et al., 2010) and removing duplicates. We will double screen at title and abstract for the first 10% of search results, including any studies we know will be included, to train the machine learning (ML) algorithm. In this review, we will take advantage of two innovative text‐mining ML capabilities of EPPI‐Reviewer 4 to reduce the initial screening workload: the priority‐screening function and the inclusion/exclusion classifier (O'Mara‐Eves et al., 2015; Thomas et al., 2011).

The priority screening function can be used at the title and abstract screening stage to prioritise the items most likely to be “included” based on previously included documents. This involves double‐screening a random test set of citations to train the priority screening function, which learns to identify relevant records based on key‐words in the title and abstract of the included and excluded studies. All core team members who are 3ie staff will be involved at this stage of screening. The function continues to learn as screening progresses. Using priority screening in this way allows for the identification of includable records at an earlier stage in the review process so that work can begin earlier on full‐text screening and data extraction. We will also use the priority screening function to classify studies into groups based on their probability of inclusion in the review. We will conduct piloting and verification of the ML functioning and expect to be able to exclude studies with <20% probability of inclusion automatically from the review. We will screen a random 10% sample of the automatically excluded studies as a check on accuracy of the function, and if all are excludable, we will auto‐exclude the rest. We will then double‐screen at title and abstract all records with likelihood of inclusion at 20% or greater.

Where a study's title and abstract do not include sufficient information to determine relevance, we will include the study for review at full text. We will double screen all studies flagged for full‐text review using two independent reviewers. We will resolve disagreements on inclusion or exclusion by discussion with a core review team member and the input of an additional core reviewer if necessary. We will assess the results of the study‐specific key‐word searches for relevance, that is, whether they cover one of the programmes included to answer our research questions and whether they provide information on the design, implementation processes, context or mechanisms at play.

#### 3.3.4 Data extraction and management

We will extract the following descriptive, methodological, qualitative and quantitative data from each included study using standardised data extraction forms (provisional forms are provided in Appendix B):

- Descriptive data including authors, publication date and status, as well as other information to characterise the study including country, type of intervention and outcome, population and context.
- Methodological information on study design, analysis method, and type of comparison (if relevant).
- Quantitative data for outcome measures, including outcome descriptive information, sample size in each intervention group, outcomes means and SDs, and test statistics (e.g., *t* test, *F* test, *p* values, 95% confidence intervals).
- Information on intervention design, including how the intervention incorporates participation, inclusion, transparency and accountability characteristics, participant adherence, contextual factors and programme mechanisms.

We will extract quantitative data for outcomes analysis using Excel. We will also extract descriptive, methodological and qualitative data using Excel. Descriptive and qualitative data will be single coded by one reviewer and checked by a second reviewer. Two independent reviewers will double code quantitative data for outcomes analysis, and any disagreement will be resolved through discussion with a third reviewer (who must be a core team member).

Once all effect sizes are calculated and converted to a standardised mean difference (SMD; as described in detail below), we will examine the data for outliers. We will define outliers as any effect sizes ±3.29 SDs from the mean, following the guidance of Tabachnick and Fidell (2001). Outliers will be windsorised as described by these authors, as is suggested for outliers in meta‐analysis (Lipsey & Wilson, 2001). Sensitivity to outliers will be examined as discussed in the section on sensitivity analysis below.

#### 3.3.5 Assessment of risk of bias in included studies

We will assess the risk of bias in the included studies by drawing on the signalling questions in the 3ie risk of bias tool, which covers both internal validity and statistical conclusion validity of experimental and quasi‐experimental impact evaluation designs (Hombrados & Waddington, 2012). It includes the bias domains and extensions to Cochrane's ROBINS‐I tool and RoB2.0 (Higgins et al., 2016; Sterne et al., 2016). The risk of bias assessment helps us to determine the extent to which the findings in each study are reliable. Two reviewers will undertake the risk of bias assessment independently. If there are disagreements, we will resolve them by discussion and the involvement of a third reviewer, as necessary. The provisional risk of bias tool can be found in Appendix C. We will do the risk of bias at the paper level, noting any potential differences in methods and risk of bias by different outcomes.

We will assess risk of bias based on the following criteria, coding each paper as “Yes”, “Probably Yes”, “Probably No”, “No” and “No Information” according to how they address each domain:

- Factors relating to baseline confounding and biases arising from differential selection into and out of the study (e.g., assignment mechanism).
- Factors relating to bias due to missing outcome data (e.g., assessment of attrition).
- Factors relating to biases due to deviations from intended interventions (e.g., performance bias and survey effects) and motivation bias (Hawthorne effects).
- Factors relating to biases in outcomes measurement (e.g., social desirability or courtesy bias, recall bias).
- Factors relating to biases in reporting of analysis.

We will report the results of the assessment for each of the assessed criteria for each study. In addition, we will use the results of the risk of bias assessments to produce an overall rating for each study as either “High risk of bias”, “Some concerns” or “Low risk of bias”, drawing on the decision rules in RoB2.0 (Higgins et al., 2016), rating studies as follows:

- “High risk of bias”: if any of the bias domains were assessed as “No” or “Probably No”.
- “Some concerns”: if one or several domains were assessed as “No Information” and none were “No” or “Probably No”.
- “Low risk of bias”: if all of the bias domains were assessed as “Yes” or “Probably Yes”.

In addition, we will attempt to explore whether there are systematic differences in outcome effects between primary studies with different risk of bias. If meta‐analysis is feasible, we will conduct sensitivity analysis to assess the robustness of the results to the risk of bias in included studies.

#### 3.3.6 Measures of treatment effect

An effect size expresses the magnitude (or strength) and direction of the relationship of interest (Borenstein et al., 2009; Valentine et al., 2015). We will extract data from each individual study to calculate standardised effect sizes for cross‐study comparison wherever possible. For continuous outcomes comparing group means in a treatment and control group, we will calculate the SMDs, or Cohen's *d*, its variance and SE using formulae provided in Borenstein et al. (2009). A SMD is a difference in means between the treatment and control groups divided by the pooled SD of the outcome measure. Cohen's *d* can be biased in cases where sample sizes are small. Therefore, in all cases we will simply adjust *d* using Hedges' method, adjusting Cohen's *d* to Hedges' *g* using the following formula (Ellis, 2010):

$$g≅d\left(1-\frac{3}{4(n_{1}+n_{2})-9}\right).$$

We will choose the appropriate formulae for effect size calculations in reference to, and dependent upon, the data provided in included studies. For example, for studies reporting means (*X*) and pooled SD for treatment (*T*) and control or comparison (*C*) at follow up only:

$$d=\frac{x_{\mathrm{Tp}+1}-x_{\mathrm{Cp}+1}}{\mathrm{SD}}.$$

If the study does not report the pooled SD, it is possible to calculate it using the following formula:

$${\mathrm{SD}}_{p+1}=\sqrt{\frac{(n_{\mathrm{Tp}+1}-1){\mathrm{SD}}_{\mathrm{Tp}+1}^{2}+(n_{\mathrm{Cp}+1}-1){\mathrm{SD}}_{\mathrm{Cp}+1}^{2}}{n_{\mathrm{Tp}+1}+n_{\mathrm{Cp}+1}-2}},$$

where the intervention is expected to change the SD of the outcome variable, we will use the SD of the control group only.

For studies reporting means ($\underline{X})$ and SDs for treatment and control or comparison groups at baseline (*p*) and follow up (*p* + 1):

$$d=\frac{{∆\underline{X}}_{p+1}-{∆\underline{X}}_{p}}{{\mathrm{SD}}_{p+1}}.$$

For studies reporting mean differences ($∆\underline{X})$ between treatment and control and SD at follow up (*p* + 1):

$$d=\frac{{∆\underline{X}}_{p+1}}{{\mathrm{SD}}_{p+1}}=\frac{{\underline{X}}_{\mathrm{Tp}+1}-{\underline{X}}_{\mathrm{Cp}+1}}{{\mathrm{SD}}_{p+1}}.$$

For studies reporting mean differences between treatment and control, SE and sample size (*n*):

$$d=\frac{{∆\underline{X}}_{p+1}}{\mathrm{SE}\sqrt{n}}.$$

As primary studies have become increasingly complex, it has become commonplace for authors to extract partial effect sizes (e.g., a regression coefficient adjusted for covariates) in the context of meta‐analysis. For studies reporting regression results, we will follow the approach suggested by Keef and Roberts (2004) using the regression coefficient and the pooled SD of the outcome. Where the pooled SD of the outcome is unavailable, we will use regression coefficients and SEs or *t* statistics to do the following, where sample size information is available in each group:

$$d=t\sqrt{\frac{1}{n_{T}}+\frac{1}{n_{C}}},$$

where *n* denotes the sample size of treatment group and control. We will use the following where only the total sample size information (*N*) is available, as suggested in Polanin et al., 2016):

$$d=\frac{2t}{\sqrt{N}}{\mathrm{Var}}_{d}=\frac{4}{N}+\frac{d^{2}}{4N}.$$

We will calculate the *t* statistic (*t*) by dividing the coefficient by the SE. If the authors only report confidence intervals and no SE, we will calculate the SE from the confidence intervals. If the study does not report the SE, but report *t,* we will extract and use this as reported by the authors. In cases in which significance levels are reported rather than *t* or SE (b), then *t* will be imputed as follows:

$$\begin{array}{cccc} & \text{Prob} & >0.1\text{:} & t=0.5,\\ 0.1\geq & \text{Prob} & >0.05\text{:} & t=1.8,\\ 0.05\geq & \text{Prob} & >0.01\text{:} & t=2.4,\\ 0.01\geq & \text{Prob:} & & t=2.8, \end{array}$$

where outcomes are reported in proportions of individuals, we will calculate the Cox‐transformed log odds ratio effect size (Sánchez‐Meca et al., 2003):

$$d=\frac{\ln(\mathrm{OR})}{1.65},$$

where OR is the odds ratio calculated from the two‐by‐two frequency table.

Where outcomes are reported based on proportions of events or days, we will use the standardised proportion difference effect size:

$$d=\frac{p_{T}-p_{C}}{\mathrm{SD}(p)},$$

where *p*_t_ is the proportion in the treatment group and *p*_c_ the proportion in the comparison group, and the denominator is given by:

$$\mathrm{SD}(p)=\sqrt{p(1-p)},$$

where p is the weighted average of *p*_c_ and *p*_t_:

$$p=\frac{n_{T}\;p_{T}+n_{C}\;p_{C}}{n_{T}+n_{C}}.$$

An independent reviewer will evaluate a random selection of 10% of effect sizes to ensure that the correct formulae were employed in effect size calculations. In all cases after synthesis, we will convert pooled effect sizes to commonly used metrics such as percentage changes and mean differences in outcome metrics typically used (e.g., weight in kg) whenever feasible.

#### 3.3.7 Unit of analysis issues

Unit of analysis errors can arise when the unit of allocation of a treatment is different to the unit of analysis of effect size estimate, and this is not accounted for in the analysis (e.g., by clustering SEs at the level of allocation). We will assess studies for unit of analysis errors (The Campbell Collaboration, 2019), and where they exist, we will correct for them by adjusting the SEs according to the following formula (Hedges, 2009; Higgins et al., 2020; Waddington et al., 2012):

$$\mathrm{SE}{\left(d\right)}^{'}=\mathrm{SE}(d)⁎\sqrt{1+(m-1)c},$$

where *m* is the average number of observations per cluster and *c* is the intra‐cluster correlation coefficient. Where included studies use robust Huber‐White SEs to correct for clustering, we will calculate the SE of *d* by dividing *d* by the *t* statistic on the coefficient of interest.

#### 3.3.8 Dealing with missing data

In cases of relevant missing or incomplete data in studies identified for inclusion, we will make every effort to contact study authors to obtain the required information. If we are unable to obtain the necessary data, we will report the characteristics of the study but state that it could not be included in the meta‐analysis or reporting of effect sizes due to missing data.

#### 3.3.9 Assessment of heterogeneity

We will assess heterogeneity by calculating the *Q* statistic, *I* ^2^, and *τ* ^2^ to provide an estimate of the amount of variability in the distribution of the true effect sizes (Borenstein et al., 2009). We will complement this with an assessment of heterogeneity of effect sizes graphically using forest plots. Additionally, we will explore heterogeneity using moderator analysis in bivariate and, where possible, multivariate meta‐regression specifications.

#### 3.3.10 Assessment of reporting biases

To reduce the possibility of publication bias, we will search for and include unpublished studies in the review. We will also test for the presence of publication bias through the use of contour‐enhanced funnel graphs (Peters et al., 2008) and statistical tests (Egger et al., 1997). Capitalising on recent shifts towards preregistration of studies and their associated preanalysis plans, we will also examine whether studies that were preregistered (e.g., on platforms such as ClinicalTrials.gov, the Open Science Foundation, the American Economic Association's trial registry, or the RIDIE) report on all of the outcomes that were proposed in their preanalysis plans. This additional analysis of outcome reporting bias may draw on methodologies used in previous work, such as the COMPare Trials Project (Goldacre et al., 2016).

#### 3.3.11 Data synthesis

We will conduct meta‐analyses of studies that we assess to be sufficiently similar. The inclusion criteria for the review are broad and we anticipate including studies that report on a diverse set of interventions, sectors and outcomes. It is therefore difficult to predict how meta‐analysis will be used in the review prospectively. However, minimum criteria will be to only combine studies using meta‐analysis when we identify two or more effect sizes using a similar outcome construct and where the comparison group state is judged to be similar across the two, similar to the approach taken by Wilson et al. (2011). We provisionally suggest that we combine studies in the same analysis when they evaluate the same intervention type, or the same outcome type. Moderator analyses can take into account multiple interventions as moderator variables, allowing us to also examine the impact of different intervention types by outcome. Where there are too few studies, or included studies are considered too heterogeneous in terms of interventions or outcomes, we will present a discussion of individual effect sizes along the causal chain. As heterogeneity exists in theory due to the variety of interventions and contexts included, we will use inverse‐variance weighted, random effects meta‐analytic models (Higgins et al., 2020).

We will use the *metafor* package (Viechtbauer, 2010) and/or the *robumeta* package (Fisher & Tipton, 2015) in R software to conduct the meta‐analyses (R Core Team, 2020).

We will conduct separate analyses for the major outcome categories: productivity, income, nutrition and health, and women's empowerment. Based on an analysis of the interventions that we find, we will attempt to further elaborate on the pathway of change that was outlined above to the extent possible. We will also use subgroup analysis to explore heterogeneity by different treatment subgroups (described in more detail in the section on subgroup analysis and investigation of heterogeneity).

We will also collect qualitative information from studies about the interventions. This information may subsequently be coded quantitatively to be used in moderator analysis. It may also be used to classify intervention mechanisms in synthesis or in the further development of intervention causal chains. These characteristics may include: intervention objectives (to change processes, behaviours or both); whether interventions are strategic (complex, adaptable strategy to realise change) or tactical (tool‐based); the source of intervention (local, NGO, government or researcher‐led); the scale of the intervention (pilot experiment vs. adoption of formal policy/law); extent to which members of both targeted groups are engaged (equally or primarily one group); and initial power differences between the groups targeted.

#### 3.3.12 Subgroup analysis and investigation of heterogeneity

Whenever feasible, we will conduct moderator analyses to investigate sources of heterogeneity. Following the PROGRESS‐PLUS approach (Olaganathan & Kar Mun, 2017), we will assess moderators falling into three broad categories of extrinsic, methodological and substantive characteristics to address inequity aspects within the aquaculture context. Examples of these categories include:

- Extrinsic characteristics: funder of the study (e.g., NGO vs. private sector vs. government investments), publication type, publication date.
- Methodological characteristics: study design, risk of bias, study quality characteristics, evaluation period, length of follow‐up.
- Substantive characteristics: participant characteristics (gender, age, socioeconomic status, education, land ownership), context (geographical setting, market access), intervention type, intervention features, type of implementing agency.

We will use random effects meta‐regression to investigate the association between moderator variables and heterogeneity of treatment effects (Borenstein et al., 2009) and subgroup analyses to investigate heterogeneity by treatment subgroups (e.g., men and women, poor and nonpoor, and so on). If the latter strategies are not possible (i.e., if we do not have sufficient number of studies or data), we will discuss and explore the factors which may be driving heterogeneity of results narratively by conducting cross‐case comparisons (Miles & Huberman, 1994).

#### 3.3.13 Sensitivity analysis

We will conduct sensitivity analysis to assess whether the results of the meta‐analysis are sensitive to the removal of any single study. We will do this by removing studies from the meta‐analysis one‐by one and assessing changes in results. We will also assess sensitivity of results to inclusion of high risk of bias studies by removing these studies from the meta‐analysis and comparing results to the main meta‐analysis results. Finally, we will assess sensitivity to outliers by comparing results with and without outliers included, as well as results when outliers are windsorised.

#### 3.3.14 Treatment of qualitative research

We will use qualitative research to supplement the findings of the interventions covered by included studies. While we will not seek out all qualitative studies relating to aquaculture activities in low‐ and middle‐income countries, we will look for qualitative studies to provide additional information about the context and implementation of interventions included in the quantitative synthesis. Specifically, this will be used to address research question 4, employing the aforementioned “effectiveness+” framework (Snilstveit, 2012). This may include feasibility studies, stakeholder analyses, formative evaluations, process evaluations, project reports, among other documents. These sources will provide key inputs to our analysis of the facilitators and inhibitors of aquaculture interventions.

We will appraise these studies and documents based on an adapted version of the Critical Appraisal Skills Programme checklist (CASP, n.d.), which is included in Appendix D. We will assess the quality of qualitative and descriptive quantitative studies by appraising the adequacy of reporting, data collection, presentation, analysis and conclusions drawn. In turn, the assessment of process evaluations will focus on sampling and methods of data collection. Finally, project documents provide information about the design or resources available for a project. As these documents provide factual information about interventions, we will not formally appraise the quality of such documents but will rather assess the relevance of the documents against the interventions included in the review.

#### 3.3.15 Treatment of cost data

To address review question 5, we will use cost data reported in the set of included studies or in additional studies identified through the second search of references. Following Shemilt et al. (2008), relevant studies will include full economic evaluations (e.g., cost‐benefit, cost‐effectiveness or cost‐utility analyses), partial economic evaluations (e.g., cost analyses, cost‐comparison studies, cost‐outcome descriptions), or any other documentation reporting cost data of included interventions.

Full and partial economic evaluation studies will be appraised in terms of the cost and/or effectiveness components reported and used in the analyses. In turn, general descriptions of cost information of included interventions will be synthesised narratively. If there is relevant data on the costs and effects of an intervention reported separately, we will extract data on the resources, unit and/or total costs with the aim to examine both components. In these cases, we will focus on comparable outcomes if possible. We will also note when included studies found statistically nonsignificant effects, however, we will not include nonsignificant impacts in the cost‐effectiveness analysis (Dhaliwal et al., 2013). If this impact is precisely measured, then there is little relevance in examining noneffective interventions; whereas if the impact is measured with less precision, there will be uncertainty around the real effectiveness of the intervention, which would affect the analysis around its cost.

## CONTRIBUTIONS OF AUTHORS

*Content*: Marta Moratti, Constanza G. Parrao, and Shannon Shisler. *Systematic review methods*: Constanza G. Parrao, Shannon Shisler, and Birte Snilstveit. *Statistical analysis*: Shannon Shisler, Constanza G. Parrao, and Marta Moratti. *Information retrieval*: John Eyers and Constanza G. Parrao.

## DECLARATIONS OF INTEREST

The authors of the review declare having no conflict of interest related to this review, they have no financial interest in it, and have not participated in previous research or publications related to the topic of this review. To minimise a potential bias in conducting the review, 3ie has two independent teams working on the impact evaluation of an aquaculture intervention and on this review, respectively. The funder and an advisory group of experts in the field have collaborated in defining the scope for the review to ensure its relevance and usability. However, they have no bearing on the implementation or reporting of this review.

## PRELIMINARY TIMEFRAME

Our tentative date for submission of the systematic review is May 2021.

## PLANS FOR UPDATING THIS REVIEW

The leading author will be responsible of updating the review 3 years after the publication of this review. If for some reason this is not possible, the leading author will communicate this to the International Development Coordinating Group.

## SOURCES OF SUPPORT

This review is part of an impact evaluation programme for an aquaculture intervention currently implemented by WorldFish, evaluated by 3ie, and funded by the Bill & Melinda Gates Foundation. The implementation and funding bodies, as well as the evaluation team, have no influence over the execution or reporting of the review.

## Footnotes

1 This review is part of a broader aquaculture impact evaluation programme conducted by 3ie and supported by the Bill & Melinda Gates Foundation. More information on the programme is available at https://www.3ieimpact.org/our-work/agriculture/impacts-aquaculture-livelihoods-nutrition-and-womens-empowerment-bangladesh.

2 We define value chain as the full range of activities that are required to bring a product or service from conception, through production and transformation, to delivery to final consumers, and final disposal after use (Kaplinsky & Morris, 2000).

3 The extent to which increased consumption comes from increases in own consumption or via higher income is an interesting research question per se. However, the effect may vary by the context, depending on which activities one is engaged with along the value chains (producing vs. nonproducing role), the type of aquaculture organisms (small vs. export‐led types), the welfare position of the household, and the accessibility and availability of aquaculture in the markets. A study on food consumption in Bangladesh (Roos, 2001) found, for example, that fish consumed from fish produced by own‐pond aquaculture only contributed 1%–11% of the total amount of fish consumed at household level, and fish sold in the markets is the single most important source of fish (57%–69%, depending on the season) for households with and without fish ponds.

4 Following van Eerdewijk et al. (2017), we refer to empowerment as the expansion of choice and strengthening of voice through the transformation of power relations, so women and girls have more control over their lives and futures.

5 This source will be used as the development database. A full example of the search strategy for this database is detailed in Appendix A.

## APPENDIX A EXAMPLE OF SEARCH STRATEGY

Database: CAB Abstracts (EBSCO)

**Table:**

S26	S24 OR S25
	4648
S25	S7 AND S18 AND S19 AND S23
	2683
S24	S4 AND S18 AND S19 AND S23
	4530
S23	S20 OR S21 OR S22
	4,269,556
S22	TI ((income* or livelihood* or production or productivity or productive or consumption or pay or payment* or earning* or remunerat* or profit* or salar* or wage or wages or expenditure or "food security" or cost‐utility or ((cost* or economic*) N3 (benefit* or effect* or evaluat*)) or (poverty N3 (reduc* or alleviat*)) or extension or training or (knowledge N4 (practis* or practic*))) OR AB ((income* or livelihood* or production or productivity or productive or consumption or pay or payment* or earning* or remunerat* or profit* or salar* or wage or wages or expenditure or "food security" or cost‐utility or ((cost* or economic*) N3 (benefit* or effect* or evaluat*)) or (poverty N3 (reduc* or alleviat*)) or extension or training or (knowledge N4 (practis* or practic*))) OR SU ((income* or livelihood* or production or productivity or productive or consumption or pay or payment* or earning* or remunerat* or profit* or salar* or wage or wages or expenditure or "food security" or cost‐utility or ((cost* or economic*) N3 (benefit* or effect* or evaluat*)) or (poverty N3 (reduc* or alleviat*)) or extension or training or (knowledge N4 (practis* or practic*)))
	1,796,348
S21	TI ((gender* or empower* or disempower* or inequit* or inequalit* or equalit* or disadvantage* or marginali* or discriminat* or vulnerab* or barrier* or "self help" or control or controlling or ownership* or (decision* N3 (make or maker* or making or made)) or confident or confidence or power* or access* or norm or norms or women or female*)) OR AB ((gender* or empower* or disempower* or inequit* or inequalit* or equalit* or disadvantage* or marginali* or discriminat* or vulnerab* or barrier* or "self help" or control or controlling or ownership* or (decision* N3 (make or maker* or making or made)) or confident or confidence or power* or access* or norm or norms or women or female*)) OR SU ((gender* or empower* or disempower* or inequit* or inequalit* or equalit* or disadvantage* or marginali* or discriminat* or vulnerab* or barrier* or "self help" or control or controlling or ownership* or (decision* N3 (make or maker* or making or made)) or confident or confidence or power* or access* or norm or norms or women or female*))
	2,718,403
S20	TI ((nutritio* or diet* or nourishment or fish‐based or "food intake" or "food consumption" or (food* N2 (varie* or divers*)) or ((eat* or consum*) N3 fish) or weight‐for‐height or weight‐for‐length or height‐for‐age or weight‐for‐age or "body mass index" or BMI or anthropometr*)) OR AB ((nutritio* or diet* or nourishment or fish‐based or "food intake" or "food consumption" or (food* N2 (varie* or divers*)) or ((eat* or consum*) N3 fish) or weight‐for‐height or weight‐for‐length or height‐for‐age or weight‐for‐age or "body mass index" or BMI or anthropometr*)) OR SU ((nutritio* or diet* or nourishment or fish‐based or "food intake" or "food consumption" or (food* N2 (varie* or divers*)) or ((eat* or consum*) N3 fish) or weight‐for‐height or weight‐for‐length or height‐for‐age or weight‐for‐age or "body mass index" or BMI or anthropometr* or "height‐weight tables"))
	924,075
S19	TI (("random* control* trial*" OR "random* trial*" OR RCT OR "cluster random* trial" OR "propensity score matching" OR PSM OR "regression discontinuity design" OR RDD OR "difference in difference*" OR "control* random* trial*" OR "case control" OR matching OR "interrupted time series" OR "random* allocation*" OR (random* N3 (allocat* OR select*)) OR "instrumental variable*" OR evaluation OR assessment OR ((quantitative OR "comparison group" OR counterfactual OR "counter factual" OR counter‐factual OR experiment*) N3 (design OR study OR analysis)) OR QED OR quasi‐experiment*)) OR AB (("random* control* trial*" OR "random* trial*" OR RCT OR "cluster random* trial" OR "propensity score matching" OR PSM OR "regression discontinuity design" OR RDD OR "difference in difference*" OR "control* random* trial*" OR "case control" OR matching OR "interrupted time series" OR "random* allocation*" OR (random* N3 (allocat* OR select*)) OR "instrumental variable*" OR evaluation OR assessment OR ((quantitative OR "comparison group" OR counterfactual OR "counter factual" OR counter‐factual OR experiment*) N3 (design OR study OR analysis)) OR QED OR quasi‐experiment*)) OR SU (("random* control* trial*" OR "random* trial*" OR RCT OR "cluster random* trial" OR "propensity score matching" OR PSM OR "regression discontinuity design" OR RDD OR "difference in difference*" OR "control* random* trial*" OR "case control" OR matching OR "interrupted time series" OR "random* allocation*" OR (random* N3 (allocat* OR select*)) OR "instrumental variable*" OR evaluation OR assessment OR ((quantitative OR "comparison group" OR counterfactual OR "counter factual" OR counter‐factual OR experiment*) N3 (design OR study OR analysis)) OR QED OR quasi‐experiment*))
	1,196,472
S18	S8 OR S9 OR S10 OR S11 OR S12 OR S13 OR S14 OR S15 OR S16 OR S17
	4,387,938
S17	GL(Afghanistan OR Albania OR Algeria OR Angola OR Antigua OR Barbuda OR Argentina OR Armenia OR Armenian OR Aruba OR Azerbaijan OR Bahrain OR Bangladesh OR Barbados OR Benin OR Belize OR Bhutan OR Bolivia OR Botswana OR Brazil OR Brasil OR "Burkina Faso" OR "Burkina Fasso" OR "Upper Volta" OR Burundi OR Urundi OR Cambodia OR "Khmer Republic" OR Kampuchea OR Cameroon OR Cameroons OR Cameron OR Camerons OR "Cape Verde" OR "Central African Republic" OR Chad OR Chile OR China OR Colombia OR Comoros OR "Comoro Islands" OR Comores OR Mayotte OR Congo OR Zaire OR "Costa Rica" OR "Cote d'Ivoire" OR "Ivory Coast" OR Cuba OR "Djibouti" OR "French Somaliland" OR Dominica OR "Dominican Republic" OR "East Timor" OR "East Timur" OR "Timor Leste" OR Ecuador OR Egypt OR "United Arab Republic" OR "El Salvador" OR Eritrea OR Ethiopia OR Fiji OR Gabon OR "Gabonese Republic" OR Gambia OR Gaza OR "Georgia Republic" OR "Georgian Republic" OR Ghana OR "Gold Coast" OR Grenada OR Guatemala OR Guinea OR Guam OR Guiana OR Guyana OR Haiti OR Honduras OR India OR Maldives OR Indonesia OR Iran OR Iraq OR Jamaica OR Jordan OR Kazakhstan OR Kazakh OR Kenya OR Kiribati OR Korea OR Kosovo OR Kyrgyzstan OR Kirghizia OR "Kyrgyz Republic" OR Kirghiz OR Kirgizstan OR "Lao PDR" OR Laos OR Lebanon OR Lesotho OR Basutoland OR Liberia OR Libya OR Madagascar OR "Malagasy Republic" OR Malaysia OR Malaya OR Malay OR Sabah OR Sarawak OR Malawi OR Nyasaland OR Mali OR "Marshall Islands" OR Mauritania OR Mauritius OR "Agalega Islands" OR Mexico OR Micronesia OR "Middle East" OR Moldova OR Moldovia OR Moldovian OR Mongolia OR Montenegro OR Morocco OR Ifni OR Mozambique OR Myanmar OR Myanma OR Burma OR Namibia OR Nepal OR Antilles OR "New Caledonia" OR Nicaragua OR Niger OR Nigeria OR "Mariana Islands" OR Oman OR Muscat OR Pakistan OR Palau OR Palestine OR Panama OR Paraguay OR Peru OR Philippines OR Philipines OR Phillipines OR Phillippines OR "Puerto Rico" OR Rwanda OR Ruanda OR "Saint Kitts" OR "St Kitts" OR Nevis OR "Saint Lucia" OR "St Lucia" OR "Saint Vincent" OR "St Vincent" OR "Grenadines" OR "Samoa" OR "Samoan Islands" OR "Navigator Island" OR "Navigator Islands" OR "Sao Tome" OR "Saudi Arabia" OR Senegal OR Seychelles OR "Sierra Leone" OR "Sri Lanka" OR "Solomon Islands" OR Somalia OR Sudan OR Suriname OR Surinam OR Swaziland OR Syria OR Tajikistan OR Tadzhikistan OR Tadjikistan OR Tadzhik OR Tanzania OR Thailand OR Togo OR "Togolese Republic" OR Tonga OR Trinidad OR Tobago OR Tunisia OR Turkey OR Turkmenistan OR Turkmen OR Uganda OR Ukraine OR Uruguay OR Uzbekistan OR Uzbek OR Vanuatu OR "New Hebrides" OR Venezuela OR Vietnam OR "Viet Nam" OR "West Bank" OR Yemen OR Zambia OR Zimbabwe OR Jamahiriya OR Jamahiryria OR Libia OR Mocambique OR Principe OR Syrian OR "Indian Ocean" OR Melanesia OR "Western Sahara")
	2,115,450
S16	TI(Afghanistan OR Albania OR Algeria OR Angola OR Antigua OR Barbuda OR Argentina OR Armenia OR Armenian OR Aruba OR Azerbaijan OR Bahrain OR Bangladesh OR Barbados OR Benin OR Belize OR Bhutan OR Bolivia OR Botswana OR Brazil OR Brasil OR "Burkina Faso" OR "Burkina Fasso" OR "Upper Volta" OR Burundi OR Urundi OR Cambodia OR "Khmer Republic" OR Kampuchea OR Cameroon OR Cameroons OR Cameron OR Camerons OR "Cape Verde" OR "Central African Republic" OR Chad OR Chile OR China OR Colombia OR Comoros OR "Comoro Islands" OR Comores OR Mayotte OR Congo OR Zaire OR "Costa Rica" OR "Cote d'Ivoire" OR "Ivory Coast" OR Cuba OR "Djibouti" OR "French Somaliland" OR Dominica OR "Dominican Republic" OR "East Timor" OR "East Timur" OR "Timor Leste" OR Ecuador OR Egypt OR "United Arab Republic" OR "El Salvador" OR Eritrea OR Ethiopia OR Fiji OR Gabon OR "Gabonese Republic" OR Gambia OR Gaza OR "Georgia Republic" OR "Georgian Republic" OR Ghana OR "Gold Coast" OR Grenada OR Guatemala OR Guinea OR Guam OR Guiana OR Guyana OR Haiti OR Honduras OR India OR Maldives OR Indonesia OR Iran OR Iraq OR Jamaica OR Jordan OR Kazakhstan OR Kazakh OR Kenya OR Kiribati OR Korea OR Kosovo OR Kyrgyzstan OR Kirghizia OR "Kyrgyz Republic" OR Kirghiz OR Kirgizstan OR "Lao PDR" OR Laos OR Lebanon OR Lesotho OR Basutoland OR Liberia OR Libya OR Madagascar OR "Malagasy Republic" OR Malaysia OR Malaya OR Malay OR Sabah OR Sarawak OR Malawi OR Nyasaland OR Mali OR "Marshall Islands" OR Mauritania OR Mauritius OR "Agalega Islands" OR Mexico OR Micronesia OR "Middle East" OR Moldova OR Moldovia OR Moldovian OR Mongolia OR Montenegro OR Morocco OR Ifni OR Mozambique OR Myanmar OR Myanma OR Burma OR Namibia OR Nepal OR Antilles OR "New Caledonia" OR Nicaragua OR Niger OR Nigeria OR "Mariana Islands" OR Oman OR Muscat OR Pakistan OR Palau OR Palestine OR Panama OR Paraguay OR Peru OR Philippines OR Philipines OR Phillipines OR Phillippines OR "Puerto Rico" OR Rwanda OR Ruanda OR "Saint Kitts" OR "St Kitts" OR Nevis OR "Saint Lucia" OR "St Lucia" OR "Saint Vincent" OR "St Vincent" OR "Grenadines" OR "Samoa" OR "Samoan Islands" OR "Navigator Island" OR "Navigator Islands" OR "Sao Tome" OR "Saudi Arabia" OR Senegal OR Seychelles OR "Sierra Leone" OR "Sri Lanka" OR "Solomon Islands" OR Somalia OR Sudan OR Suriname OR Surinam OR Swaziland OR Syria OR Tajikistan OR Tadzhikistan OR Tadjikistan OR Tadzhik OR Tanzania OR Thailand OR Togo OR "Togolese Republic" OR Tonga OR Trinidad OR Tobago OR Tunisia OR Turkey OR Turkmenistan OR Turkmen OR Uganda OR Ukraine OR Uruguay OR Uzbekistan OR Uzbek OR Vanuatu OR "New Hebrides" OR Venezuela OR Vietnam OR "Viet Nam" OR "West Bank" OR Yemen OR Zambia OR Zimbabwe OR Jamahiriya OR Jamahiryria OR Libia OR Mocambique OR Principe OR Syrian OR "Indian Ocean" OR Melanesia OR "Western Sahara")
	781,645
S15	AB(Afghanistan OR Albania OR Algeria OR Angola OR Antigua OR Barbuda OR Argentina OR Armenia OR Armenian OR Aruba OR Azerbaijan OR Bahrain OR Bangladesh OR Barbados OR Benin OR Belize OR Bhutan OR Bolivia OR Botswana OR Brazil OR Brasil OR "Burkina Faso" OR "Burkina Fasso" OR "Upper Volta" OR Burundi OR Urundi OR Cambodia OR "Khmer Republic" OR Kampuchea OR Cameroon OR Cameroons OR Cameron OR Camerons OR "Cape Verde" OR "Central African Republic" OR Chad OR Chile OR China OR Colombia OR Comoros OR "Comoro Islands" OR Comores OR Mayotte OR Congo OR Zaire OR "Costa Rica" OR "Cote d'Ivoire" OR "Ivory Coast" OR Cuba OR "Djibouti" OR "French Somaliland" OR Dominica OR "Dominican Republic" OR "East Timor" OR "East Timur" OR "Timor Leste" OR Ecuador OR Egypt OR "United Arab Republic" OR "El Salvador" OR Eritrea OR Ethiopia OR Fiji OR Gabon OR "Gabonese Republic" OR Gambia OR Gaza OR "Georgia Republic" OR "Georgian Republic" OR Ghana OR "Gold Coast" OR Grenada OR Guatemala OR Guinea OR Guam OR Guiana OR Guyana OR Haiti OR Honduras OR India OR Maldives OR Indonesia OR Iran OR Iraq OR Jamaica OR Jordan OR Kazakhstan OR Kazakh OR Kenya OR Kiribati OR Korea OR Kosovo OR Kyrgyzstan OR Kirghizia OR "Kyrgyz Republic" OR Kirghiz OR Kirgizstan OR "Lao PDR" OR Laos OR Lebanon OR Lesotho OR Basutoland OR Liberia OR Libya OR Madagascar OR "Malagasy Republic" OR Malaysia OR Malaya OR Malay OR Sabah OR Sarawak OR Malawi OR Nyasaland OR Mali OR "Marshall Islands" OR Mauritania OR Mauritius OR "Agalega Islands" OR Mexico OR Micronesia OR "Middle East" OR Moldova OR Moldovia OR Moldovian OR Mongolia OR Montenegro OR Morocco OR Ifni OR Mozambique OR Myanmar OR Myanma OR Burma OR Namibia OR Nepal OR Antilles OR "New Caledonia" OR Nicaragua OR Niger OR Nigeria OR "Mariana Islands" OR Oman OR Muscat OR Pakistan OR Palau OR Palestine OR Panama OR Paraguay OR Peru OR Philippines OR Philipines OR Phillipines OR Phillippines OR "Puerto Rico" OR Rwanda OR Ruanda OR "Saint Kitts" OR "St Kitts" OR Nevis OR "Saint Lucia" OR "St Lucia" OR "Saint Vincent" OR "St Vincent" OR "Grenadines" OR "Samoa" OR "Samoan Islands" OR "Navigator Island" OR "Navigator Islands" OR "Sao Tome" OR "Saudi Arabia" OR Senegal OR Seychelles OR "Sierra Leone" OR "Sri Lanka" OR "Solomon Islands" OR Somalia OR Sudan OR Suriname OR Surinam OR Swaziland OR Syria OR Tajikistan OR Tadzhikistan OR Tadjikistan OR Tadzhik OR Tanzania OR Thailand OR Togo OR "Togolese Republic" OR Tonga OR Trinidad OR Tobago OR Tunisia OR Turkey OR Turkmenistan OR Turkmen OR Uganda OR Ukraine OR Uruguay OR Uzbekistan OR Uzbek OR Vanuatu OR "New Hebrides" OR Venezuela OR Vietnam OR "Viet Nam" OR "West Bank" OR Yemen OR Zambia OR Zimbabwe OR Jamahiriya OR Jamahiryria OR Libia OR Mocambique OR Principe OR Syrian OR "Indian Ocean" OR Melanesia OR "Western Sahara")
	1,417,604
S14	TI ((developing or less* N1 developed or "under developed" or underdeveloped or "middle income" or low* N1 income or underserved or "under served" or deprived or poor*) N1 (countr* or nation* or population* or world)) OR AB ((developing or less* N1 developed or "under developed" or underdeveloped or "middle income" or low* N1 income or underserved or "under served" or deprived or poor*) N1 (countr* or nation* or population* or world)) OR SU ((developing or less* N1 developed or "under developed" or underdeveloped or "middle income" or low* N1 income or underserved or "under served" or deprived or poor*) N1 (countr* or nation* or population* or world))
	4,191,490
S13	TI ((developing or less* N1 developed or "under developed" or underdeveloped or "middle income" or low* N1 income) N1 (economy or economies)) OR AB ((developing or less* N1 developed or "under developed" or underdeveloped or "middle income" or low* N1 income) N1 (economy or economies)) OR SU ((developing or less* N1 developed or "under developed" or underdeveloped or "middle income" or low* N1 income) N1 (economy or economies))
	2310
S12	TI (low* N1 (gdp or gnp or "gross domestic" or "gross national")) OR AB (low* N1 (gdp or gnp or "gross domestic" or "gross national")) OR SU (low* N1 (gdp or gnp or "gross domestic" or "gross national"))
	160
S11	TI (low 3 middle N3 countr*) OR AB (low 3 middle N3 countr*) OR SU (low 3 middle N3 countr*)
	9
S10	TI ((lmic or lmics or "third world" or "lami country" or "lami countries")) OR AB ((lmic or lmics or "third world" or "lami country" or "lami countries")) OR SU ((lmic or lmics or "third world" or "lami country" or "lami countries"))
	43,978
S9	TI (("transitional country" or "transitional countries")) OR AB (("transitional country" or "transitional countries")) OR SU (("transitional country" or "transitional countries"))
	144
S8	TI (Africa or Asia or Caribbean or "West Indies" or "South America" or "Latin America" or "Central America") OR AB (Africa or Asia or Caribbean or "West Indies" or "South America" or "Latin America" or "Central America") OR SU (Africa or Asia or Caribbean or "West Indies" or "South America" or "Latin America" or "Central America") OR GL (Africa or Asia or Caribbean or "West Indies" or "South America" or "Latin America" or "Central America")
	2,404,838
S7	S5 OR S6
	46,203
S6	DE "salmon culture" or DE "frog culture" or DE "turtle culture"
	890
S5	TI (((fish* or tilapia or carp or shrimp or mussel* or shellfish or crustacean* or mollusc* or rice‐fish or frog* or turtle* or seaweed) N2 ((farm* or culture or small‐scale or pond or pond* or cage*))) OR AB (((fish* or tilapia or carp or shrimp or mussel* or shellfish or crustacean* or mollusc* or rice‐fish or frog* or turtle* or seaweed) N2 ((farm* or culture or small‐scale or pond or pond* or cage*))) OR SU (((fish* or tilapia or carp or shrimp or mussel* or shellfish or crustacean* or mollusc* or rice‐fish or frog* or turtle* or seaweed) N2 ((farm* or culture or small‐scale or pond or pond* or cage*))
	46,203
S4	S1 OR S2 OR S3
	143,695
S3	CC "MM120"
	131,344
S2	DE "aquaculture" OR DE "brackishwater aquaculture" OR DE "fish culture" OR DE "freshwater aquaculture" OR DE "marine aquaculture" OR DE "agropisciculture" OR DE "shellfish culture" OR DE "wastewater aquaculture" OR DE "growout ponds" OR DE "fish production"
	60,077
S1	TI ((aquaculture or ((fish* or shellfish or rice‐fish or seaweed) N3 (farm* or culture or small‐scale or cage*)) or "pond culture" or polyculture or fishpond* or Mallahin or fisherwomen or pisciculture)) OR AB ((aquaculture or ((fish* or shellfish or rice‐fish or seaweed) N3 (farm* or culture or small‐scale or cage*)) or "pond culture" or polyculture or fishpond* or Mallahin or fisherwomen or pisciculture))
	48,289

## APPENDIX B DATA EXTRACTION TOOLS

The following tables provide provisional data extraction tools for descriptive, qualitative, and effect size data. If necessary, we could amend these tools to better capture key characteristics of primary studies.

**Table B1:** Provisional tool for descriptive and qualitative data coding

	Description	Question	Coding
Report identify‐cation	Unique study identification #		For example, AQC001
	First author—impact evaluation	Surname	Surname
	Other papers used for coding	First author surname and type of paper of any qualitative, descriptive quantitative, process evaluations, used for coding	
	Publication date	Year (letter—if more than one study from that author and that year)	XXXX (a)
	Publication type	What is the impact evaluation publication type?	1 = Peer‐reviewed journal 2 = Book chapter/book 3 = Conference paper 4 = Organisation report 5 = Working paper 6 = Implementation document 7 = Other grey 8 = PhD thesis/dissertation
	Funding agency	Who is funding the evaluation/study?	1 = Public institution (e.g., govt, university, research institute) 2 = Private institution (e.g., private company) 3 = Multilateral Organisation (World Bank, UN) 4 = Foundations 5 = NGO 8 = Not clear 9 = Not applicable (nonfunded)
	Name of funding agency	Please add name of the agency funding the evaluation	Open answer
	Independence of evaluation	What level of independence is there between the implementing agency and study team?	1 = Funding and author team independent of implementers/funders of programme 2 = Funding independent of implementers/funders of programme, but includes authors from funder/implementer 3 = Evaluation funded and undertaken by funders/implementers 8 = Unclear
	Independent data collection	Has the data been collected by an independent party?	1 = Yes, 2 = No, 8 = Not clear
	Conflict of interest	Is there a potential conflict of interest associated with study which could influence results collected/reported? (e.g., Is there a declaration of conflict of interest? Is any of the authors related in any way to the funding or implementing institution?)	1 = Yes, 2 = No, 8 = Not clear
	Comments on conflict of interest	Please add reason for your answer to whether there is a conflict of interest	Open answer
	Language of publication	Language of publication of the impact evaluation, for example, Spanish, English, and so forth	Open answer
	Other methods	If the impact evaluation addresses other questions than effectiveness note questions and methods used here	Open answer (this will include, for example, mixed‐methods to assess implementation, adherence, participant views, etc.)
Context	Country	List countries the study was conducted in	Country 1, Country 2, and so forth
	Detailed location	If provided, give detailed information on where the study took place within a country, for example regions/districts covered	Open answer
	World Bank Region	Select region(s) the study was conducted in according to World Bank. For more info on region classification see http://data.worldbank.org/country	1 = East Asia & Pacific 2 = Europe & Central Asia 3 = Latin America & Caribbean 4 = Middle East & North Africa 5 = South Asia 6 = Sub‐Saharan Africa
	WB income category	Select the World Bank income classification of the country at the time of the study	1 = Low income country 2 = Lower‐middle income country 3 = Upper‐middle income country
	Programme or project name	State the programme or project name. If no name, then list the location	Open answer
Intervention descriptives	Intervention type	Write a short paragraph to describe the intervention type and characteristics. The description should be as much detailed as possible	Open answer The codes will be added later once the interventions categories are defined
	Objectives of intervention	State any objectives stated in study or other document	Open answer
	Program theory	Do the authors make explicit reference to program theory, theory of change or similar?	1 = Yes, 2 = No, 8 = Not clear
		Report any description/statement of program theory as stated by author(s)	Open answer
		Is the study using theory to inform the evaluation design and analysis?	Open answer—describe if and how the authors use theory in the evaluation. Do they for example use it to inform data collection? Do they do any causal chain analysis?
	Intervention development	To what extent is the intervention locally developed/demanded or donor created? Please present any information presented in the paper(s), N/A if no information presented	Open answer
	Intervention implementing agency	Who is implementing the intervention? State the name (and department) of the implementing agency	Open answer
	Intervention funding agency	Type of funder	1 = Government 2 = NGO 3 = Multilateral/bilateral organisation 4 = Foundation 5 = Private sector 6 = Other
	Intervention funding agency	Name of intervention funding agency	Open answer
	Intervention target group	What were the characteristics of beneficiaries used to target the intervention?	Open answer
	Targeting methods	How were beneficiaries targeted for the programme (e.g., how was the targeting implemented)?	Open answer
	Intervention start	Start date (if not stated, state study date) of intervention	XX/XXXX
	Intervention end	State end date (if ongoing state ongoing)	XX/XXXX
	Intervention length	Start intervention length (months)	State number of months
	Consideration of equity	Does the study consider equity?	1 = Yes, 2 = No
	Equity methods	How does the study consider equity?	1 = Does not address gender or equity 2 = Vulnerable population targeted 3 = Sub‐group analysis by sex 4 = Sub‐group analysis (other than sex) 5 = Equity sensitive analytical framework 6 = Equity sensitive methodology 7 = Equity sensitive research process 8 = Measures effects on an inequality outcome 9 = Ethics approval referenced 10 = Ethics informed by equity
	Equity dimension	What dimension(s) of equity does the study consider? PROGRESS + indicators (multiple choice—may pick more than one)	1 = Age (e.g., old or young age) 2 = Conflict‐affected 3 = Culture (includes language) 4 = Disability (medical, physical, neurological, mental disorders) 5 = Education 6 = Ethnicity 7 = Head of household (female or male) 8 = Land size 9 = Land ownership 10 = Place of residence (rural, urban, peri‐urban, etc.) 11 = Religion 12 = Socioeconomic status (income or poverty status) 13 = Social capital 14 = Sex (includes the use of the term gender meaning the biological sex of a person) 15 = Other (vulnerable group not typified by any of the above) 16 = Not applicable
Process and implementation	Information about program take‐up	Is there any information about program take‐up? Commentary by authors should be used when information on program take/up, and so forth, is not backed up by some sort of research/when the authors do not report that/how they collected data to assess these areas.	1 = Yes, commentary from author; 2 = Yes, formally assessed, 3 = No
	Methods of assessing take‐up	Which methods are used to assess program take‐up?	1 = Observation by intervention staff 2 = Reporting by participants 3 = Other 4 = Commentary from author 9 = Not measured
	Results of the assessment of take‐up	What is the result/information provided of the assessment of program take‐up?	Open answer
	Information about program adherence (among beneficiaries)	Is there any information about program adherence (among beneficiaries)? Commentary by authors should be used when information on program adherence, and so forth, is not backed up by some sort of research/when the authors do not report that/how they collected data to assess these areas	1 = Yes, commentary from author; 2 = Yes, formally assessed, 3 = No
	Methods of assessing adherence	Which methods are used to assess program adherence?	1 = Observation by intervention staff 2 = Reporting by participants 3 = Other 4 = Commentary from author 9 = Not measured
	Results of the assessment of adherence	What is the result/information provided of the assessment of program adherence?	Open answer
	Information about implementation fidelity/intervention delivery quality	Is there any information on implementation fidelity/intervention delivery quality? Commentary by authors should be used when information on program adherence, and so forth, is not backed up by some sort of research/when the authors do not report that/how they collected data to assess these areas	1 = Yes, commentary from author; 2 = Yes, formally assessed, 3 = No
	Methods of assessing intervention fidelity	Which methods are used to assess implementation fidelity/intervention delivery quality	1 = Observation by intervention staff 2 = Reporting by participants 3 = Other 4 = Commentary from author 9 = Not measured
	Results of the assessment of intervention fidelity	What is the result/information provided of the assessment of implementation fidelity/intervention delivery quality	Open answer
	Other description of process/implementation factors	Any other description of process/implementation factors not covered above	Open answer
	Causal mechanisms/barriers and enablers	Does the study identify any causal mechanisms/barriers and enablers related to context (not included above)?	1 = Yes, commentary from author 2 = Yes, formally assessed 3 = No
	Methods	How are these identified?	1 = Observation by intervention staff 2 = Reporting by participants 3 = Other 4 = Commentary from author
	Results	Report here any material relevant to causal mechanisms and barriers and enablers	Open answer
Cost data	Cost	Are any unit cost data/cost‐effectiveness estimates provided?	1 = Yes, 2 = No
	Cost details	If yes, report any details of unit cost and/or total cost. Please also report year and currency	Open answer
External validity	Efficacy or effectiveness trial	Was the intervention implemented under "real world" conditions? By real world we mean a programme implemented independently of the evaluation, either by government, NGO or international agency	1 = Yes, 2 = No, 9 = N/A
	Sampling frame for the study	State the sampling frame (list of all those within a population who can be sampled, that is, households, communities) for selection of study participants (i.e., Census, etc.)	Open answer
	Author discussion of external validity	Do the authors discuss or explicitly address generalisability/applicability?	Open answer

**Table B2:** Provisional Tool For Effect Size Data Coding

	Description	Question	Coding
ID	Unique study identification #		For example, AQC001
	Unique effect size identification #		For example, AQC001_01, AQC001_02, AQC001_03, and so forth
	First author—impact evaluation	Surname	Open answer
Outcome for effect size (answer for all studies)	Outcome	Which outcome is being coded?	1 = Productivity 2 = Income 3 = Nutrition or health 4 = Empowerment 5 = Other (specify)
	Definition of outcome	Please provide the authors definition of the outcome (including description of the subgroup if relevant)	Open answer
	Exposure to intervention (in months)	How long is the intervention exposure itself?	#
	Evaluation period (in months)	The total number of months elapsed between offering an intervention and the point at which an outcome measure is taken post intervention, or as a follow‐up measurement. If <1 month, use decimals (e.g., 1 week would be 0.25)	#
	Comparison	What type of comparison group is used?	1 = No intervention (service delivery as usual) 2 = Other intervention 3 = Pipeline (wait‐list) control (still service intervention the delivery as usual)
	Describe Comparison Group	If answer above is (1) no intervention, type N/A, if (2) Other Intervention, list what control group is receiving, if (3) Pipeline control, report when the control group will receive the intervention in relation to the treatment group (e.g., 1 year later)	Free Text
	Counterfactual	How is the counterfactual chosen?	Free text (e.g., random control trial, propensity score matching, etc.)—Multiple codes are ok
	Subgroup analysis	Is this effect size data for a subgroup?	0 = No 1 = Yes
	Subgroup analysis description	If yes to question 2, which type of subgroup?	Open answer—this can include separate samples for gender, income, place of residence
	Source	Which page(s) contain the effect size data?	Open answer
	Data to be extracted	Which type of data to be extracted?	1 = Continuous—means and SDs 2 = Continuous—mean difference and SD 3 = Dichotomous outcome—proportions 4 = Regression data—dichotomous outcome (e.g., logistic regression) 5 = Regression data—continuous outcome (e.g., linear regression)
	Analysis type for this effect size	What type of analysis was used (Regression, 2SLS, ANCOVA, etc.)? Multiple codes are ok	Free text
Effect size data (answer for all studies)	Sample size metric	Sample size unit of analysis	1 = Individual 2 = Household 3 = Group (e.g., community org) 4 = Village 5 = Other 6 = Not clear
	Treatment effect estimated	What treatment effect is estimated?	1 = ITT 2 = ATET 3 = ATE 4 = LATE
	Sample size (treatment)	Initial sample size treatment group	#
	Sample size (control)	Initial sample size control group	#
	Sample size (total)	Initial sample size total	#
	Observations (treatment)	Number of treatment observations after attrition/follow up	#
	Observations (control)	Number of control observations after attrition/follow up	#
	Observations (total)	Total number of control observations after attrition/follow up	#
Outcome data—if continuous (Means and SDs)	Baseline outcome treatment	State result of baseline outcome for treatment group	#
	SD Baseline outcome treatment	State SD of baseline outcome measure for treatment group	#
	Baseline outcome control	State result of baseline outcome for control group	#
	SD Baseline outcome control	State SD of baseline outcome measure for control group	#
	Outcome in treatment post intervention	State result of post intervention outcome for treatment group	#
	SD Outcome in treatment post intervention	State SD of post intervention outcome measure for treatment group	#
	Outcome in control post intervention	State result of post intervention outcome for control group	#
	SD Outcome in control post intervention	State SD of post intervention outcome measure for control group	#
	Outcome in treatment 1st follow up	State result of 1st follow up outcome measure for treatment group	#
	SD Outcome in treatment 1st follow up	State SD 1st follow up outcome measure for treatment group	#
	Outcome in control 1st follow up	State result of 1st follow up outcome measure for treatment group	#
	SD Outcome in control 1st follow up	State SD 1st follow up outcome measure for treatment group	#
Outcome data—If continuous (Mean difference and SD/SE at follow up)	Mean difference at follow up	State mean difference	#
	SD at follow up	State SD at follow up	#
	SE	State SE	#
Outcomes data—if dichotomous (Proportions r)	Baseline number with outcome in treatment	State result of baseline outcome for treatment group	#
	Proportion with outcome at baseline in treatment	State proportion with outcome at baseline in treatment	#
	Baseline number with outcome in control	State result of baseline outcome for treatment group	#
	Proportion with outcome at baseline in control	State proportion with outcome at baseline in control	#
	Number with outcome in treatment post intervention	State number with outcome post intervention for treatment group	#
	Proportion with outcome in treatment group post intervention	State proportion with outcome post intervention in control group	#
	Number with outcome in control post intervention	State number with outcome post intervention for control group	#
	Proportion with outcome in control group post intervention	State proportion with outcome post intervention in control group	#
	Number with outcome in treatment 1st follow up	State number with outcome at 1st follow up for treatment group	#
	Proportion with outcome in treatment group 1st follow up	State proportion with outcome at 1st follow up in treatment group	#
	Number with outcome in control 1st follow up	State number with outcome at 1st follow up for control group	#
	Proportion with outcome in control group 1st follow up	State proportion with outcome at 1st follow up in control group	#
Regression data	Type of coefficient	What is the coefficient type?	1 = raw 2 = standardised 3 = other
	Coefficient	What is the coefficient estimate?	#
	Pooled SD of outcome	What is the pooled SD of the outcome?	#
	SE	What is the SE of the coefficient estimate?	#
	*t* test	What is the *t* statistic associated with the focal predictor?	#
	*p* value	What is the *p* value associated with the coded effect?	#

## APPENDIX C RISK OF BIAS ASSESSMENT TOOL

The following table provides a provisional tool to guide the risk of bias assessment for quantitative impact evaluations. If necessary, we could amend the tool to better inform the appraisal of primary studies.

**Table C1:** Provisional risk of bias assessment tool

	Description	Question	Coding
ID	Unique study identification #	Study	For example, AQC001
	Paper	Surname/year of first author of paper for effect size data extraction	Open answer
Research methods—study design and risk of bias	Design type	What type of study design is used?	1 = RCT (random assignment to households/individuals) or quasi‐RCT 2 = Cluster‐RCT (quasi‐RCT) 3 = Natural experiment: randomised or as‐if randomised 4 = Natural experiment: regression discontinuity (RD) 5 = CBA (nonrandomised assignment with treatment and contemporaneous comparison group, baseline and endline data collection)—individual repeated measurement 6 = CBA pseudo panel (repeated measurement for groups but different individuals) 7 = Interrupted time series (with or without contemporaneous control group) 8 = Panel data, but no baseline (pretest) 9 = Comparison group with endline data only
	Methods used for analysis	Which methods are used to control for selection bias and confounding?	1 = Statistical matching (PSM, CEM, covariate matching) 2 = Difference in differences (DID) estimation methods 3 = IV‐regression (2‐stage least squares or bivariate probit) 4=Heckman selection model 5 = Fixed effects regression 6 = Covariate adjusted estimation 7 = Propensity weighted regression 8 = Comparison of means 9 = Other
	Design and analysis method description	Briefly describe the study design and analysis method undertaken by the authors	Open answer
	Unit of analysis	Is unit of analysis in cluster allocation addressed in standard error calculation (RCT and NRS)?	1 = Yes 2 = No 3 = Not reported/unclear 4 = Not applicable
	Method used to address differences between UoA and unit of data collection	Briefly describe methods used to adjust standard errors to account for correlation of observations within clusters (e.g., cluster‐robust standard errors reported)	Open answer
	Type of comparison group	Indicate type of comparison group	1 = No intervention (service delivery as usual) 2 = Other PITA intervention 3 = Pipeline (wait‐list) control (still service delivery as usual)
	Assignment mechanism	(1) Mechanism of assignment: was the allocation or identification mechanism random or as good as random?	1 = Yes 2 = Probably yes 3 = Probably no 4 = No 8 = No information/unclear
	Assignment justification	Justification for coding decision (Include a brief summary of justification for rating, mentioning your response to all sub questions, cite relevant pages)	Open answer
	Confounding	Group equivalence: was the method of analysis executed adequately to ensure comparability of groups throughout the study and prevent confounding	1 = Yes 2 = Probably yes 3 = Probably no 4 = No 8 = No information/unclear
	Confounding justification	Justification for coding decision (Include a brief summary of justification for rating, mentioning your response to all sub questions, cite relevant pages)	Open answer
	Selection bias	Was any differential selection into or out of the study (attrition bias) adequately resolved?	1 = Yes 2 = Probably yes 3 = Probably no 4 = No 8 = No information/unclear
	Selection bias justification	Justification for coding decision (include a brief summary of justification for rating, mentioning your response to all sub questions, cite relevant pages)	Open answer
	Spill‐overs, cross‐overs and contamination	(2) Spill‐overs, cross‐overs and contamination: was the study adequately protected against spill‐overs, cross‐overs and contamination?	1 = Yes, 2 = Probably yes, 3 = Probably no, 4 = No, 8 = No information/unclear
	Spill‐overs justification	Justification for coding decision (Include a brief summary of justification for rating, mentioning your response to all sub questions, cite relevant pages)	Open answer
	Motivation bias	Was the process of being observed free from motivation bias (e.g., Hawthorne effects)?	1 = Yes 2 = Probably Yes 3 = Probably No 4 = No 8 = No Information/Unclear
	Motivation justification	Justification for coding decision (Include a brief summary of justification for rating, mentioning your response to all sub questions, cite relevant pages)	Open answer
	Outcome reporting	(3) Outcome reporting: was the study free from selective outcome reporting?	1 = Yes 2 = Probably Yes 3 = Probably No 4 = No 8 = No Information/Unclear
	Outcome reporting	Justification for coding decision (Include a brief summary of justification for rating, mentioning your response to all sub questions, cite relevant pages)	Open answer
	Analysis reporting	4: Analysis reporting: was the study free from selective analysis reporting?	1 = Yes 2 = Probably Yes 3 = Probably No 4 = No 8 = No Information/Unclear
	Analysis reporting	Justification for coding decision (Include a brief summary of justification for rating, mentioning your response to all sub questions, cite relevant pages)	Open answer
	Performance bias	(5) Performance bias: was the process of being observed free from motivation bias?	1 = Yes 2 = Probably Yes 3 = Probably No 4 = No 8 = No Information/Unclear
	Performance bias	Justification for coding decision (Include a brief summary of justification for rating, mentioning your response to all sub questions, cite relevant pages)	Open answer
	Other bias	(6) Other risks of bias: is the study free from other sources of bias? Including around measurement of the intervention	1 = Yes 2 = Probably Yes 3 = Probably No 4 = No 8 = No Information/Unclear
	Other bias	Justification for coding decision (include a brief summary of justification for rating, mentioning your response to all sub questions, cite relevant pages)	Open answer
	Blinded participants	Blinding of participants?	1 = Yes 2 = No 9 = N/A
	Blinded observers	Blinding of outcome assessors?	1 = Yes 2 = No 9 = N/A
	Blinded analysts	Blinding of data analysts?	1 = Yes 2 = No 9 = N/A
	Method used to blind	Describe method(s) used to blind	Open answer (including describe method of placebo control)

## APPENDIX D CRITICAL APPRAISAL OF QUALITATIVE STUDIES TOOL

The following table provides a provisional critical appraisal tool for qualitative and descriptive quantitative studies. If necessary, we could amend the tool to better capture key characteristics of primary studies.

**Table D1:** Provisional critical appraisal tool for qualitative studies

Critical appraisal of qualitative and descriptive quantitative studies
1. Is the research aim clearly stated? (Yes/No)
Reporting:
2. Description of the context? (Yes/No)
3. Description of sampling procedures? (Yes/No)
4. Are sample characteristics sufficiently reported? (sample size, location, and at least one additional characteristic) (Yes/No)
5. Is it clear how the data were collected (e.g., for interviews, is there an indication of how interviews were conducted? (Yes/No)
6. Methods of recording of data reported? (Yes/No)
7. Methods of analysis explicitly stated? (Yes/No)
Methodology:
8. Is there a clear link to relevant literature/theoretical framework? (Yes/No)
9. Is the design appropriate to answer the research question? (Yes/No)
10. Was the sampling strategy appropriate to the aims of the research? (Yes/No)
11. Were the data collected in a way that addressed the research issue? (Yes/No)
12. Was the data analysis sufficiently rigorous? (Yes/No)
13. Has triangulation been applied? (Yes/No)
14. Is the analysis and conclusions clearly presented? (Yes/No)
15. Does the paper discuss ethical considerations related to the research? (Yes/No)

## References

[cl21188-bib-0001] Ahmed, M. , & Lorica, M. H. (2002). Improving developing country food security through aquaculture development—Lessons from Asia. Food Policy, 27, 125–141. 10.1016/S0306-9192(02)00007-6

[cl21188-bib-0002] Ahmed, M. , Rab, M. A. , & Bimbao, M. A. P. (1995). *Aquaculture technology adoption in Kapasia Thana, Bangladesh: Some preliminary results from farm record‐keeping data* (43p No. 44). International Center for Living Aquatic Resources Management, Manila, Philippines.

[cl21188-bib-0003] Akber, M. A. , Aziz, A. A. , & Lovelock, C. (2020). Major drivers of coastal aquaculture expansion in Southeast Asia. Ocean & Coastal Management, 198, 105364. 10.1016/j.ocecoaman.2020.105364

[cl21188-bib-0004] Barbier, E. B. , Hacker, S. D. , Kennedy, C. , Koch, E. W. , Stier, A. C. , & Silliman, B. R. (2011). The value of estuarine and coastal ecosystem services. Ecological Monographs, 81, 169–193. 10.1890/10-1510.1

[cl21188-bib-0005] Bird, F. A. , Pradhan, A. , Bhavani, R. V. , & Dangour, A. D. (2019). Interventions in agriculture for nutrition outcomes: A systematic review focused on South Asia. Food Policy, 82, 39–49. 10.1016/j.foodpol.2018.10.015

[cl21188-bib-0006] Borenstein, M. , Hedges, L. V. , Higgins, J. P. T. , & Rothstein, H. R. (2009). Introduction to meta‐analysis. John Wiley & Sons.

[cl21188-bib-0007] Brummett, R. E. , Gockowski, J. , Pouomogne, V. , & Muir, J. (2011). Targeting agricultural research and extension for food security and poverty alleviation: A case study of fish farming in Central Cameroon. Food Policy, 36, 805–814. 10.1016/j.foodpol.2011.07.012

[cl21188-bib-0008] CASP , n.d. *Critical appraisal skills programme checklists* [WWW Document]. https://casp-uk.net/casp-tools-checklists/

[cl21188-bib-0009] CORDIS . (2015). *Final report summary—AFSPAN (Aquaculture for Food Security, Poverty Alleviation and Nutrition)*. https://cordis.europa.eu/project/id/289760/reporting

[cl21188-bib-0010] D'Armengol, L. , Prieto Castillo, M. , Ruiz‐Mallén, I. , & Corbera, E. (2018). A systematic review of co‐managed small‐scale fisheries: Social diversity and adaptive management improve outcomes. Global Environmental Change, 52, 212–225. 10.1016/j.gloenvcha.2018.07.009

[cl21188-bib-0011] De La Rue, L. , Polanin, J. R. , Espelage, D. L. , & Pigott, T. D. (2013). PROTOCOL: School‐based interventions to reduce dating and sexual violence: A systematic review. Campbell Systematic Reviews, 9, 1–43. 10.1002/CL2.106

[cl21188-bib-0012] Dey, M. M. , & Ahmed, M. (2005). Aquaculture—Food and livelihoods for the poor in Asia: A brief overview of the issues. Aquaculture Economics & Management, 9, 1–10. 10.1080/13657300591004970

[cl21188-bib-0013] Dhaliwal, I. , Duflo, E. , Glennerster, R. , & Tulloch, C. (2013). Comparative cost‐effectiveness analysis to inform policy in developing countries: A general framework with applications for education. In P. Glewwe (Ed.), Education policy in developing countries. University of Chicago Press. 10.7208/chicago/9780226078854.001.0001

[cl21188-bib-0014] Edwards, P. (2000). Aquaculture, poverty impacts and livelihoods. Natural Resource Perspectives, 56, 4.

[cl21188-bib-0015] Egger, M. , Smith, G. D. , Schneider, M. , & Minder, C. (1997). Bias in meta‐analysis detected by a simple, graphical test. British Medical Journal, 315, 629–634. 10.1136/bmj.315.7109.629 9310563 PMC2127453

[cl21188-bib-0016] Ellis, P. D. (2010). The essential guide to effect sizes: Statistical power, meta‐analysis, and the interpretation of research results. Cambridge University Press.

[cl21188-bib-0017] FAO . (2014a). The state of world fisheries and aquaculture: Opportunities and challenges. Food and Agriculture Organization of the United Nations.

[cl21188-bib-0018] FAO . (2014b). *Sustainable fisheries and aquaculture for food security and nutrition*. A report by the High Level Panel of Experts on Food Security and Nutrition of the Committee on World Food Security. Food and Agriculture Organization of the United Nations, Rome.

[cl21188-bib-0019] FAO . (2020a). FAO yearbook. Fishery and aquaculture statistics 2018. Food and Agriculture Organization of the United Nations.

[cl21188-bib-0020] FAO . (2020b). The state of world fisheries and aquaculture: Sustainability in action. Food and Agriculture Organization of the United Nations.

[cl21188-bib-0021] Fisher, Z. , & Tipton, E. (2015). robumeta: An R‐package for robust variance estimation in meta‐analysis. arXiv.

[cl21188-bib-0022] Gambelli, D. , Vairo, D. , Solfanelli, F. , & Zanoli, R. (2019). Economic performance of organic aquaculture: A systematic review. Marine Policy, 108, 103542. 10.1016/j.marpol.2019.103542

[cl21188-bib-0023] GEP , n.d. *Project database*. The Global Environment Facility. https://www.thegef.org/projects

[cl21188-bib-0024] Goldacre, B. , Drysdale, H. , Powell‐Smith, A. , Dale, A. , Milosevic, I. , Slade, E. , Hartley, P. , Marston, C. , Mahtani, K. , & Heneghan, C. (2016). *The COMPare Trials Project*. COMPare. http://compare-trials.org 10.1186/s13063-019-3173-2 PMC6375128 30760329

[cl21188-bib-0025] Halwart, M. , Martinez, M. , & Schückler, A. (2000). *Small ponds make a big difference: Integrating fish with crop and livestock farming*. FAO.

[cl21188-bib-0026] Haque, A. B. M. M. , & Dey, M. M. (2017). Impacts of community‐based fish culture in seasonal floodplains on income, food security and employment in Bangladesh. Food Section, 9, 25–38. 10.1007/s12571-016-0629-z

[cl21188-bib-0027] Harper, S. , Zeller, D. , Hauzer, M. , Pauly, D. , & Sumaila, U. R. (2013). Women and fisheries: Contribution to food security and local economies. Marine Policy, 39, 56–63. 10.1016/j.marpol.2012.10.018

[cl21188-bib-0028] Hawkes, C. , & Ruel, M. (2006). The links between agriculture and health: An intersectoral opportunity to improve the health and livelihoods of the poor. Bulletin of the World Health Organization, 84, 984–990. 17242835 10.2471/blt.05.025650 PMC2627573

[cl21188-bib-0029] Hedges, L. (2009). Effect sizes in nested designs. In H. Cooper , L. Hedges , & J. Valentine (Eds.), The handbook of research synthesis and meta‐analysis (2nd ed., pp. 337–355). Russell Sage Foundation.

[cl21188-bib-0030] Hedges, L. V. , Tipton, E. , & Johnson, M. C. (2010). Robust variance estimation in meta‐regression with dependent effect size estimates. Research Synthesis Methods, 1, 39–65. 10.1002/jrsm.5 26056092

[cl21188-bib-0031] Henriksson, P. J. G. , Tran, N. , Mohan, C. V. , Chan, C. Y. , Rodriguez, U. P. , Suri, S. , Mateos, L. D. , Utomo, N. B. P. , Hall, S. , & Phillips, M. J. (2017). Indonesian aquaculture futures—Evaluating environmental and socioeconomic potentials and limitations. Journal of Cleaner Production, 162, 1482–1490. 10.1016/j.jclepro.2017.06.133

[cl21188-bib-0032] Higgins, J. , Thomas, J. , Chandler, J. , Cumpston, M. , Li, T. , Page, M. & Welch, V. (Eds.), (2020). *Cochrane handbook for systematic reviews of interventions*, version 6.1 (updated September 2020). Cochrane. 10.1002/14651858.ED000142 PMC10284251 31643080

[cl21188-bib-0033] Higgins, J. P. , Sterne, J. A. , Savovic, J. , Page, M. J. , Hróbjartsson, A. , Boutron, I. , Reeves, B. , & Eldridge, S. (2016). A revised tool for assessing risk of bias in randomized trials. The Cochrane Database of Systematic Reviews, 10, 29–31.

[cl21188-bib-0034] Hombrados, G. J. , & Waddington, H. (2012). *Internal validity in social experiments and quasi‐experiments: An assessment tool for reviewers* (Working Paper). 3ie.

[cl21188-bib-0035] IFPRI . (2012). *Women's empowerment in agriculture index*. International Food Policy Research Institute, Oxford Poverty and Human Development Initiative, Feed the Future.

[cl21188-bib-0036] Johnson, N. , Balagamwala, M. , Pinkstaff, C. , Theis, S. , Meinzen‐Dick, R. , & Quisumbing, A. (2018). *How do agricultural development projects empower women? Linking strategies with expected outcomes. How do agricultural development projects empower women? Linking strategies with expected outcomes*. 10.19268/JGAFS.322018.1

[cl21188-bib-0037] Johnson, N. L. , Kovarik, C. , Meinzen‐Dick, R. , Njuki, J. , & Quisumbing, A. (2016). Gender, assets, and agricultural development: Lessons from eight projects. World development, 83, 295–311. 10.1016/j.worlddev.2016.01.009 31007355 PMC6472297

[cl21188-bib-0038] Kaplinsky, R. , & Morris, M. (2000). *A handbook for value chain research*. IDRC.

[cl21188-bib-0039] Kassam, L. , & Dorward, A. (2017). A comparative assessment of the poverty impacts of pond and cage aquaculture in Ghana. Aquaculture, 470, 110–122. 10.1016/j.aquaculture.2016.12.017

[cl21188-bib-0040] Kawarazuka, N. , 2010. The contribution of fish intake, aquaculture, and small‐scale fisheries to improving nutrition: A literature review (Working Paper No. 2106). The WorldFish Center, Penang, Malaysia.

[cl21188-bib-0041] Keef, S. , & Roberts, L. (2004). The meta‐analysis of partial effect sizes. The British Journal of Mathematical and Statistical Psychology, 57, 97–129. 10.1348/000711004849303 15171803

[cl21188-bib-0042] Keus, E. , Subasinghe, R. , Aleem, N. , Sarwer, R. , Islam, M. , Hossain, M. , Masum, A. , Rahman, M. M. , Alan, M. , Anisuzzaman, A. , Bhuiyan, M. , Rahman, M. F. , & Bhuiya, M. (2017). *Aquaculture for income and nutrition: Final report*. Program Report: 2017‐30. WorldFish, Penang, Malaysia.

[cl21188-bib-0043] Khondker, M.‐E.‐J. , Ahmed, M. , & Belton, B. (2010). The impacts of aquaculture development on food security: Lessons from Bangladesh. Aquaculture Research, 41, 481–495. 10.1111/j.1365-2109.2009.02337.x

[cl21188-bib-0044] Koh, H. , Teh, S. , Kh'ng, X. , & Raja Barizan, R. (2018). Mangrove forests: Protection against and resilience to coastal disturbances. JTFS, 30, 446–460. 10.26525/jtfs2018.30.5.446460

[cl21188-bib-0045] Kruijssen, F. , Albert, J. A. , Morgan, M. , Boso, D. , Siota, F. , Sibiti, S. , & Schwarz, A. J. (2013). *Livelihoods, markets, and gender roles in Solomon Islands: Case studies from Western and Isabel Provinces* (Research Program on Aquatic Agricultural Systems), Project Report: AAS‐2013‐22. CGIAR, Penang, Malaysia.

[cl21188-bib-0046] Kruijssen, F. , McDougall, C. L. , & van Asseldonk, I. J. M. (2018). Gender and aquaculture value chains: A review of key issues and implications for research. Aquaculture, 493, 328–337. 10.1016/j.aquaculture.2017.12.038

[cl21188-bib-0047] Kruijssen, F. , Rajaratnam, S. , Choudhury, A. , McDougall, C. , & Dalsgaard, J. (2016). *Gender in the farmed fish value chain of Bangladesh: A review of the evidence and development approaches* (Program Brief: 2016‐38). The WorldFish Center, Penang, Malaysia.

[cl21188-bib-0048] Leroy, J. L. , & Frongillo, E. A. (2007). Can interventions to promote animal production ameliorate undernutrition? Journal of Nutrition, 137, 2311–2316. 10.1093/jn/137.10.2311 17885016

[cl21188-bib-0049] Lipsey, M. W. , & Wilson, D. B. (2001). Practical meta‐analysis. Applied social research methods series (Vol. 49). Sage.

[cl21188-bib-0050] Macdonald, G. , Higgins, J. P. , Ramchandani, P. , Valentine, J. C. , Bronger, L. P. , Klein, P. , O'Daniel, R. , Pickering, M. , Rademaker, B. , Richardson, G. , & Taylor, M. (2012). Cognitive‐behavioural interventions for children who have been sexually abused: A systematic review. Campbell Systematic Reviews, 8, 1–111. 10.4073/csr.2012.14 PMC7061273 22592679

[cl21188-bib-0051] Masset, E. , Haddad, L. , Cornelius, A. , & Isaza‐Castro, J. (2012). Effectiveness of agricultural interventions that aim to improve nutritional status of children: Systematic review. British Medical Journal, 344, d8222. 10.1136/bmj.d8222 22251864 PMC3259800

[cl21188-bib-0052] Mcleod, E. , Chmura, G. L. , Bouillon, S. , Salm, R. , Björk, M. , Duarte, C. M. , Lovelock, C. E. , Schlesinger, W. H. , & Silliman, B. R. (2011). A blueprint for blue carbon: Toward an improved understanding of the role of vegetated coastal habitats in sequestering CO 2. Frontiers in Ecology and the Environment, 9, 552–560. 10.1890/110004

[cl21188-bib-0053] Miles, M. B. , & Huberman, A. M. (1994). An expanded source book: Qualitative data analysis (2nd ed.). SAGE Publications.

[cl21188-bib-0054] Mohamed, N. , & Dodson, B. (1998). Sustainable rural livelihoods? Evaluating the potential of small‐scale aquaculture in the Western Cape. Development Southern Africa, 15, 103–121. 10.1080/03768359808439998

[cl21188-bib-0055] Morgan, M. , Terry, G. , Rajaratnam, S. , & Pant, J. (2017). Socio‐cultural dynamics shaping the potential of aquaculture to deliver development outcomes. Rev Aquacult, 9, 317–325. 10.1111/raq.12137

[cl21188-bib-0056] Ndanga, L. Z. B. , Quagrainie, K. K. , & Dennis, J. H. (2013). Economically feasible options for increased women participation in Kenyan aquaculture value chain. Aquaculture, 414–415, 183–190. 10.1016/j.aquaculture.2013.08.012

[cl21188-bib-0057] O'Mara‐Eves, A. , Thomas, J. , McNaught, J. , Miwa, M. , & Ananiadou, S. (2015). Using text mining for study identification in systematic reviews: A systematic review of current approaches. Systematic Reviews, 4, 5. 10.1186/2046-4053-4-5 25588314 PMC4320539

[cl21188-bib-0058] Olaganathan, R. , & Kar Mun, A. T. (2017). Impact of aquaculture on the livelihoods and food security of rural communities. International Journal of Fisheries and Aquatic Studies, 5, 278–283.

[cl21188-bib-0059] Oliver, S. , Dickson, K. , Bangpan, M. , & Newman, M. (2017). Getting started with a review, An Introduction to Systematic Reviews (pp. 71–92). SAGE Publications.

[cl21188-bib-0060] Pant, J. , Barman, B. K. , Khondker, M.‐E.‐J. , Belton, B. , & Beveridge, M. (2014). Can aquaculture benefit the extreme poor? A case study of landless and socially marginalized Adivasi (ethnic) communities in Bangladesh. Aquaculture, 418–419, 1–10. 10.1016/j.aquaculture.2013.09.027

[cl21188-bib-0061] Peters, J. L. , Sutton, A. J. , Jones, D. R. , Abrams, K. R. , & Rushton, L. (2008). Contour‐enhanced meta‐analysis funnel plots help distinguish publication bias from other causes of asymmetry. Journal of Clinical Epidemiology, 61, 991–996. 10.1016/j.jclinepi.2007.11.010 18538991

[cl21188-bib-0062] Phillips, M. , Subasinghe, R. , Tran, N. , Kassam, L. , & Chan, C. Y. (2016). *Aquaculture big numbers* (No. 601). FAO Fisheries and Aquaculture Technical Paper. FAO, FAO Rome.

[cl21188-bib-0063] Polanin, J. R. , Tanner‐Smith, E. E. , & Hennessy, E. A. (2016). Estimating the difference between published and unpublished effect sizes: A meta‐review. Review of educational research, 86, 207–236. 10.3102/0034654315582067

[cl21188-bib-0064] Pullin, R. & Shehadeh, Z. (Eds.) (1980). *Integrated agriculture‐aquaculture farming systems*. Conference Proceedings 4. Presented at the International Center for Living Aquatic Resources Management, Manila, Philippines, p. 258.

[cl21188-bib-0065] Quisumbing, A. , Roy, S. , Njuki, J. , Tanvin, K. , & Waithanji, E. (2013). *Can dairy value‐chain projects change gender norms in rural Bangladesh? Impacts on assets, gender norms, and time use* (Discussion Paper No. 01311), Poverty, Health, and Nutrition Division. International Food Policy Research Institute (IFPRI), Washington, D.C.

[cl21188-bib-0066] R Core Team . (2020). *R: A language and environment for statistical computing*. R Foundation for Statistical Computing, Vienna, Austria. https://www.r-project.org/

[cl21188-bib-0067] Ramírez, E. , & Ruben, R. (2015). Gender systems and women's labor force participation in the salmon industry in Chiloé, Chile. World Development, 73, 96–104. 10.1016/j.worlddev.2014.11.003

[cl21188-bib-0068] Rand, J. , & Tarp, F. (2009). Impact of an aquaculture extension project in Bangladesh. Journal of Development Effectiveness, 1, 130–146. 10.1080/19439340902918110

[cl21188-bib-0069] Rashid, S. , Minot, N. , & Lemma, S. (2019). Does a “Blue Revolution” help the poor? Evidence from Bangladesh. Agricultural Economics, 50, 139–150. 10.1111/agec.12472

[cl21188-bib-0070] Reale, P. , & Phillips, M. (2020). SDG14—A holistic approach to sustainable development. In S. Nicklin , B. Cornwell , & L. Trowbridge (Eds.), A better world: Life below water (pp. 14–19). Human Development Forum.

[cl21188-bib-0071] Richards, D. R. , & Friess, D. A. (2016). Rates and drivers of mangrove deforestation in Southeast Asia, 2000–2012. Proceedings of the National Academy of Sciences of the United States of America, 113, 344–349. 10.1073/pnas.1510272113 26712025 PMC4720307

[cl21188-bib-0072] Roos, N. (2001). *Fish consumption and aquaculture in rural Bangladesh: Nutritional contribution and production potential of culturing small indigenous fish species (SIS) in pond polyculture with commonly cultured carps* (Ph.D.). The Royal Veterinary and Agricultural University, Denmark.

[cl21188-bib-0073] Ruel, M. T. , & Alderman, H. (2013). Nutrition‐sensitive interventions and programmes: How can they help to accelerate progress in improving maternal and child nutrition? The Lancet, 382, 536–551. 10.1016/S0140-6736(13)60843-0 23746780

[cl21188-bib-0074] Ruel, M. T. , Quisumbing, A. R. , & Balagamwala, M. (2018). Nutrition‐sensitive agriculture: What have we learned so far? Global Food Security, 17, 128–153. 10.1016/j.gfs.2018.01.002

[cl21188-bib-0075] Saiful Islam, A. H. M. , Barman, B. K. , & Khondker, M.‐E.‐J. (2015). Adoption and impact of integrated rice–fish farming system in Bangladesh. Aquaculture, 447, 76–85. 10.1016/j.aquaculture.2015.01.006

[cl21188-bib-0076] Sánchez‐Meca, J. , Marín‐Martínez, F. , & Chacón‐Moscoso, S. (2003). Effect‐size indices for dichotomized outcomes in meta‐analysis. Psychological Methods, 8, 448–467. 10.1037/1082-989X.8.4.448 14664682

[cl21188-bib-0077] Santos, F. , Fletschner, D. , Savath, V. , & Peterman, A. (2014). Can government‐allocated land contribute to food security? Intrahousehold analysis of West Bengal's microplot allocation program. World Development, 64, 860–872. 10.1016/j.worlddev.2014.07.017

[cl21188-bib-0078] Sari, I. , McDougall, C. , Rajaratnam, S. , & Park, C. M. Y. (2017). Women's empowerment in aquaculture: Two case studies from Indonesia. FAO, World Fish.

[cl21188-bib-0079] Shemilt, I. , Mugford, M. , Byford, S. , Drummond, M. , Eisenstein, E. , Knapp, M. , Mallender, J. , Marsh, K. , McDaid, D. , Vale, L. , & Walker, D. (2008). *The Campbell collaboration economics methods policy brief*. Campbell Collaboration.

[cl21188-bib-0080] Snilstveit, B. (2012). Systematic reviews: From “bare bones” reviews to policy relevance. Journal of Development Effectiveness, 4, 388–408. 10.1080/19439342.2012.709875

[cl21188-bib-0081] Sterne, J. A. , Hernán, M. A. , Reeves, B. C. , Savović, J. , Berkman, N. D. , Viswanathan, M. , Henry, D. , Altman, D. G. , Ansari, M. T. , Boutron, I. , Carpenter, J. R. , Chan, A.‐W. , Churchill, R. , Deeks, J. J. , Hróbjartsson, A. , Kirkham, J. , Jüni, P. , Loke, Y. K. , Pigott, T. D. , … Higgins, J. P. (2016). ROBINS‐I: A tool for assessing risk of bias in non‐randomised studies of interventions. British Medical Journal, 355, 4919. 10.1136/bmj.i4919 PMC5062054 27733354

[cl21188-bib-0082] Swindale, A. , & Bilinksy, P. (2006). *Household Dietary Diversity Score (HDDS) for measurement of household food access: Indicator guide* (Version 2). Food and Nutrition Technical Assistance III Project (FANTA). USAID.

[cl21188-bib-0083] Tabachnick, B. G. , & Fidell, L. S. (2001). Using multivariate statistics (4th ed.). Allyn & Bacon.

[cl21188-bib-0084] The Campbell Collaboration . (2019). *Campbell systematic reviews: Policies and guidelines* (Version 1.4), Campbell Policies and Guidelines Series No. 1.

[cl21188-bib-0085] The Methods Group of the Campbell Collaboration . (2019a). *Methodological expectations of Campbell Collaboration intervention reviews: Conduct standards*. Campbell Policies and Guidelines Series No. 3.

[cl21188-bib-0086] The Methods Group of the Campbell Collaboration . 2019b. Methodological expectations of Campbell Collaboration intervention reviews: Reporting standards. Campbell Policies and Guidelines Series No. 4.

[cl21188-bib-0087] Thomas, J. , Brunton, J. , & Graziosi, S. (2010). *EPPI‐Reviewer 4.0: Software for research synthesis*. EPPI‐Centre Software. Social Science Research Unit, UCL Institute of Education, London.

[cl21188-bib-0088] Thomas, J. , McNaught, J. , & Ananiadou, S. (2011). Applications of text mining within systematic reviews. Research Synthesis Methods, 2, 1–14. 10.1002/jrsm.27 26061596

[cl21188-bib-0089] Toufique, K. A. , & Belton, B. (2014). Is aquaculture pro‐poor? Empirical evidence of impacts on fish consumption in Bangladesh. World Development, 64, 609–620.

[cl21188-bib-0090] USAID . (2013). *Greater Harvest and Economic Returns from Shrimp (GHERS)*. Final Program Performance Report. United States Agency for International Development, Bangladesh.

[cl21188-bib-0091] Valentine, J. C. , Aloe, A. M. , & Lau, T. S. (2015). Life after NHST: How to describe your data without “p‐ing” everywhere. Basic and Applied Social Psychology, 37, 260–273. 10.1080/01973533.2015.1060240

[cl21188-bib-0092] Valiela, I. , Bowen, J. L. , & York, J. K. (2001). Mangrove forests: One of the world's threatened major tropical environments. BioScience, 51, 807. 10.1641/0006-3568(2001)051[0807:MFOOTW]2.0.CO;2

[cl21188-bib-0093] van Eerdewijk, A. , Wong, F. , Vaast, C. , Newton, J. , Tyszler, M. , & Pennington, A. (2017). White paper: A conceptual model of women and girls' empowerment. Royal Tropical Institute (KIT).

[cl21188-bib-0094] Veliu, A. , Gessese, N. , Ragasa, C. , & Okali, C. (2009). *Gender analysis of aquaculture value chain in Northeast Vietnam and Nigeria* (Agriculture and rural development discussion paper No. 44). World Bank Group, Washington, D.C.

[cl21188-bib-0095] Viechtbauer, W. (2010). Conducting meta‐analyses in R with the metafor package. Journal of Statistical Software, 36, 1–48. 10.18637/jss.v036.i03

[cl21188-bib-0096] Waddington, H. , Aloe, A. , Becker, B. , Djimeu, E. W. , Hombrados, J. G. , Tugwell, P. , Wells, G. , & Reeves, B. (2017). Quasi‐experimental study designs series—paper 6: Risk of bias assessment. Journal of Clinical Epidemiology, 89, 43–52. 10.1016/j.jclinepi.2017.02.015 28351693

[cl21188-bib-0097] Waddington, H. , White, H. , Snilstveit, B. , Hombrados, J. G. , Vojtkova, M. , Davies, P. , Bhavsar, A. , Eyers, J. , Koehlmoos, T. P. , Petticrew, M. , Valentine, J. C. , & Tugwell, P. (2012). How to do a good systematic review of effects in international development: A tool kit. Journal of Development Effectiveness, 4, 359–387. 10.1080/19439342.2012.711765

[cl21188-bib-0098] Weeratunge, N. , Snyder, K. A. , & Sze, C. P. (2010). Gleaner, fisher, trader, processor: Understanding gendered employment in fisheries and aquaculture. Fish and Fisheries, 11, 405–420. 10.1111/j.1467-2979.2010.00368.x

[cl21188-bib-0099] Wilson, D. B. , Weisburd, D. , & McClure, D. (2011). Use of DNA testing in police investigative work for increasing offender identification, arrest, conviction and case clearance. Campbell Systematic Reviews, 7, 1–53. 10.4073/csr.2011.7

[cl21188-bib-0100] World Bank . (2007). *From agriculture to nutrition: Pathways, synergies and outcomes*. The International Bank for Reconstruction and Development/The World Bank.

[cl21188-bib-0101] World Bank . (2013). *Fish to 2030: Prospects for fisheries and aquaculture, agriculture and environmental services* (Discussion Paper 3).

[cl21188-bib-0102] World Bank . (n.d.). *World Bank country and lending groups*. https://datahelpdesk.worldbank.org/knowledgebase/articles/906519-world-bank-country-and-lending-groups

[cl21188-bib-0103] WorldFish . (n.d.). Feed the future Bangladesh aquaculture and nutrition activity. https://www.worldfishcenter.org/content/feed-future-bangladesh-aquaculture-and-nutrition-activity

